# Uncovering Potential Applications of Cyanobacteria and Algal Metabolites in Biology, Agriculture and Medicine: Current Status and Future Prospects

**DOI:** 10.3389/fmicb.2017.00515

**Published:** 2017-04-25

**Authors:** Rachana Singh, Parul Parihar, Madhulika Singh, Andrzej Bajguz, Jitendra Kumar, Samiksha Singh, Vijay P. Singh, Sheo M. Prasad

**Affiliations:** ^1^Ranjan Plant Physiology and Biochemistry Laboratory, Department of Botany, University of AllahabadAllahabad, India; ^2^Faculty of Biology and Chemistry, Institute of Biology, University of BialystokBialystok, Poland; ^3^Department of Botany, Govt. Ramanuj Pratap Singhdev Post-Graduate CollegeBaikunthpur, Koriya, India

**Keywords:** algae, biofuel, cyanobacteria, cyanotoxins, food products, pharmaceuticals

## Abstract

Cyanobacteria and algae having complex photosynthetic systems can channelize absorbed solar energy into other forms of energy for production of food and metabolites. In addition, they are promising biocatalysts and can be used in the field of “white biotechnology” for enhancing the sustainable production of food, metabolites, and green energy sources such as biodiesel. In this review, an endeavor has been made to uncover the significance of various metabolites like phenolics, phytoene/terpenoids, phytols, sterols, free fatty acids, photoprotective compounds (MAAs, scytonemin, carotenoids, polysaccharides, halogenated compounds, etc.), phytohormones, cyanotoxins, biocides (algaecides, herbicides, and insecticides) etc. Apart from this, the importance of these metabolites as antibiotics, immunosuppressant, anticancer, antiviral, anti-inflammatory agent has also been discussed. Metabolites obtained from cyanobacteria and algae have several biotechnological, industrial, pharmaceutical, and cosmetic uses which have also been discussed in this review along with the emerging technology of their harvesting for enhancing the production of compounds like bioethanol, biofuel etc. at commercial level. In later sections, we have discussed genetically modified organisms and metabolite production from them. We have also briefly discussed the concept of bioprocessing highlighting the functioning of companies engaged in metabolites production as well as their cost effectiveness and challenges that are being addressed by these companies.

## Introduction

The world population, which accounted six billion in 1999 rose to seven billion in 2011, and is estimated to touch upto nine billion by 2050. With over increasing population, the need for resources is also increasing, which in turn increases our dependency on agricultural crops (Guihéneuf et al., 2016). However, even after over-utilization of agricultural crops for food, chemicals, and biofuels, the need of growing population has not been fulfilled. Taking into account the challenges, which are rising due to mismanagement in food and energy resources, a question arises: where we will land in the upcoming future? That is why the necessity of addressing these challenges has raised.

**Figure 1:**
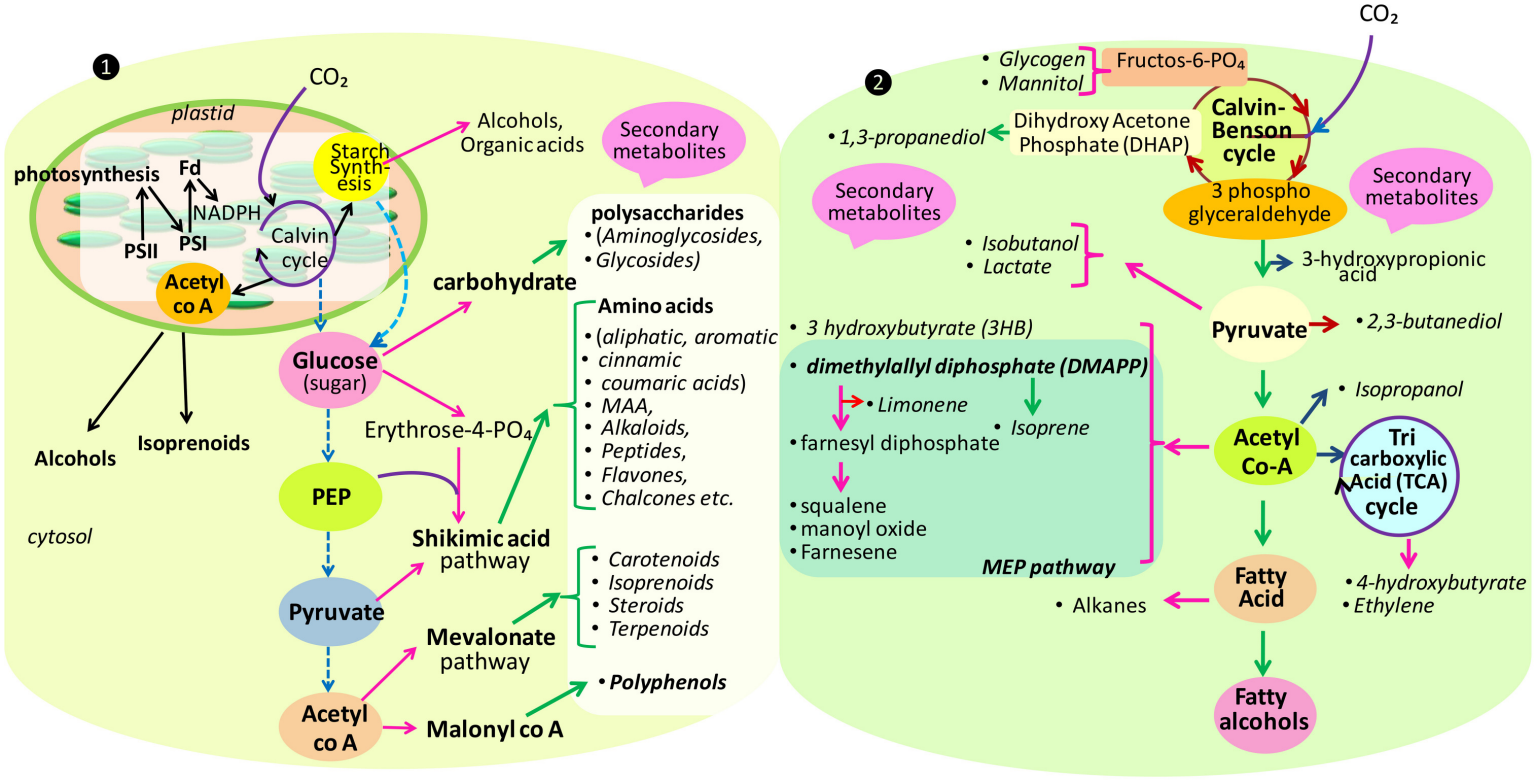
**Main pathways for the biosynthesis of some secondary as well-primary (carbohydrate, starch, alcohol, etc.) metabolites in eukaryote (❶)** and prokaryote **(**❷**)** (modified after Burja et al., 2001; Oliver et al., 2016).

To achieve future food demands, cyanobacteria and algae have presented themselves as the most promising candidates because they are endowed with the complex photosynthetic system (Mulkidjanian et al., 2006), and can absorb a broad wavelength of the solar radiation for channelizing this energy into other chemicals (Furukawa et al., 2006; Chisti, 2007; Pisciotta et al., 2010). Another aspect which makes them more suitable is that they do not require arable lands for their growth. They can grow on residual nutrients with high productivity along with an enrichment in lipids (60–65% of dry weight), proteins, total fibers (33–50% higher than higher plants) and carbohydrates, which could cut out the high prices of food obtained from agriculture-based industries (Rittmann, 2008; Guihéneuf et al., 2016). Cyanobacteria and algae are the immense sources of several metabolites such as alkaloids, carbohydrates, flavanoids, pigments, phenols, saponins, steroids, tannins, terpenes, and vitamins which can be utilized in biotechnology and industrial fields (Guihéneuf et al., 2016). Some metabolites such as cyanotoxins are reported to have toxic effects, but they can be exploited for their allelochemical nature and can be introduced in agricultural fields as pesticides i.e., algicides, fungicides, weedicides, and herbicides. Apart from the toxic metabolite production, they are also enriched with several pharmacologically active compounds that have antibacterial (Volka and Furkert, 2006; Malathi et al., 2014), anticancerous (Gerwick et al., 1994; Mukund and Sivasubramanian, 2014; Semary and Fouda, 2015), antifungal (Rath and Priyadarshani, 2013; Shaieb et al., 2014), antiplasmodial (Papendorf et al., 1998), antiviral (Patterson et al., 1994; Abdo et al., 2012), and immunosuppressive (Koehn et al., 1992; Vijayakumar and Menakha, 2015) activities, which have aggravated interest in cyanobacterial and algal secondary metabolites. Thus, due to high pharmaceutical values, a new perspective of utilizing cyanobacteria and algae in the field of medicine has risen. The pathways utilized by these organisms for metabolite productions are different (Figure 1). For instance, mevalonate pathway is involved in isoprenoids synthesis in algae, but in case of prokaryotes, they are synthesized by non-mevalonate pathway.

In this article, we have summarized functions of various metabolites like phenolics, phytoene/terpenoids, phytols, sterols, free fatty acids, photoprotective compounds (MAAs, scytonemin, carotenoids, polysaccharides, halogenated compounds etc.), phytohormones and toxins obtained from cyanobacteria and algae. In addition, we have briefly mentioned usefulness of bioengineering for enhancing the production of metabolites which paves the way for their commercialization. Also we have focused on the concept of bioprocessing and new technologies that are being currently used. Besides, we have also discussed role of cyanobacteria and algae in the production of biomedicine and biofuel. In later sections, the role of algal biomass organization involved in commercializing these products has been also discussed along with the limitations and the productivity costs.

## An outlook of metabolites: basically what they are?

### Phenolics

**Figure 2:**
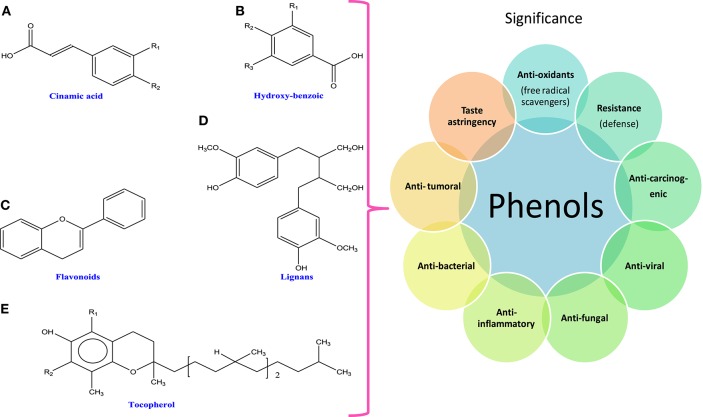
**Chemical structures and significance of polyphenols: (A)** cinamic acid, **(B)** hydroxy-benzoic acid, **(C)** flavonoids, **(D)** lignans, and **(E)** tocopherols obtained from different cyanobacteria and algae.

Polyphenols are the group of secondary metabolites such as phenolic acids, flavonoids (flavanones, flavonols, chalcones, flavones, flavan-3-ols, and flavanonols), tannins, and lignins (Thomas and Kim, 2011; Figure 2). Among different phenolic compounds, phlorotannins (eight interconnected flavonoid rings) are the group of tannins and phloroglucinols (Wang et al., 2012) that have been isolated from brown algae (15% of dry weight; Le Gall et al., 2015). These phlorotannins are reported to have antioxidant activities in the biological system (Ferrari et al., 2015; Gómez et al., 2016). Moreover, phenolics are characterized as stress compounds, which participate in defense mechanisms against biotic stresses like grazing (Coleman et al., 2007), settlement of bacteria (Lau and Qian, 2000), and abiotic stresses like UV irradiation (Coba et al., 2009) and metal toxicity (Connan and Stengel, 2011). Secondary metabolites are not directly involved in growth processes but some reports have suggested the participation of phlorotannins in regulating developmental processes in brown algae (Schoenwaelder and Wiencke, 2000; Gómez et al., 2016). Structurally, phenolic compounds have at least one phenolic ring and show strong biological activities, when halogenated (Cabrita et al., 2010). Metabolites like phytoalexins, lignin, flavonoids, furanocoumarins, tannins, and anthocyanins are involved in the defense system of the algae and cyanobacteria against adverse conditions (Adeyemi, 2011; Stengel et al., 2011). In addition, *Microcystis aeruginosa*, a cyanobacterium has been found to have inhibitory effects on growth due to the presence of polyphenols such as ellagic and gallic acids and catechin (Nakai et al., 2005). Kumar et al. (2008) have shown that main compounds of phlorotannins group are fucols, phlorethols, fucophlorethols, fuhalols, halogenated, and sulfated phlorotannins that have great potential under oxidative stress and also these compounds are capable of curing diseases caused by free radicals. Similarly, other phenolic compounds like catechin, epigallocatechin gallate, catechol, rutin, morin, caffeic acid, and hesperidin isolated from red algae have been found to exhibit anti-inflammatory activity (Ibànez and Cifuentes, 2013; Guihéneuf et al., 2016). Earlier, researchers have shown anticarcinogenic, antiviral, antibacterial, antifungal, anti-inflammatory, and antitumoral properties of cyanobacteria and algae that were attributed to the presence of novel compounds such as antioxidants, phycobilins, phenols, polysaccharides, steroids, and terpenoids (Munawer and Mazharuddin, 2011; Chauhan and Kasture, 2014; Kumar et al., 2016; Table 1).

**Table 1:** **An overview of metabolites from cyanobacteria and algae and their potential uses**.

**Algae/cyanobacteria**	**Metabolites**	**Uses**	**References**
*Arthrospira platensis, Nostoc muscorum, Phormidium foveolarum*, and *Spirulina platensis*	Phenolic compounds	Potentially considered for pharmaceutical and nutritional uses (for example as additive in the preparation of functional food). Prevents vascular damage as well as cardiovascular diseases progression. Provides defense by scavenging the free radicals. Acts as a UV-B screening compound, provide resistance to plants against pathogens, pests, and diseases.	Rice-Evans et al., 1997; Singh et al., 2003; Camera et al., 2004; Vogt, 2010; Ferrari et al., 2015; Kumar et al., 2016
*Synechocystis* sp., *Anabaena, Nostoc, Spirulina, Phaeodactylum tricornutum, P. lutheri*, and *Nostoc commune*	Fatty acids	Highly potent to be used as liquid transport fuels. Consume as in diet due to high content of polyunsaturated fatty acid, protein, and vitamins. Provides chemical defense as it is toxic to grazers. Lipid extracts may be used as a herbal medicine, to treat against cancer, viruses, burns, and chronic fatigue.	Anupama and Ravindra, 2000; Jüttner, 2001; Rasmussen et al., 2008; Guedes et al., 2011; Hellier et al., 2013
*Synechocystis* sp.	Terpenoids	Highly potent to be used as hydrocarbon biofuel. Provides chemical defense against herbivory, fragrances, and flavors.	Kirby and Keasling, 2009; Bentley et al., 2013
*Anabaena doliolum* and *Scytonema javanicum*	Mycosporine-glycine, Porphyra-334, Shinorine	Provides protection against UV-B, high temperature, and photooxidative stress. Acts as osmolytes and improves antioxidant status, which subsequently lowers the level of ROS.	Oren and Gunde-Cimerman, 2007; Klisch and Häder, 2008; Singh et al., 2010
*Nostoc muscorum, Phormidium foveolarum*, and *Spirulina platensis*	Carotenoids, β-Carotene, Lutein, Zeaxanthin, Cryptoxanthin, α-carotene, Lycopene	Protects PSII, light harvesting complexes, and reaction center. Improve antioxidant status of the organisms. Use in food industry. Use to prevent cancer.	Cardozo et al., 2007; Prasanna et al., 2010; Kumar et al., 2016
*Scytonema*	Scytonemin	Having an unique pharmacological potential and used as anti-inflammatory and antiproliferative agent.	Stevenson et al., 2002
Chondrus ocellatus^*^	Carragenans, Agar, and Lectins	Widely used as antitumor, antiviral, anticoagulant, and immunomodulation agent. Used as a vehicle to deliver drugs.	Marinho-Soriano and Bourret, 2003; Jepson et al., 2004; Cardozo et al., 2007
*Synechococcus elongates PCC7942, Cylindrospermopsis raciborskii 339-T3, Fischerella, Microcystis aeruginosa NPCD-1*, and *Microcystis panniformis SCP702*	Halogenated compounds	Exhibit antiviral, antifungal, antifouling, antiproliferative, antibacterial, anti-inflammatory activity. Show cytotoxic, antifeedant, insecticidal, and ichthyotoxic responses.	Blunt et al., 2009; Rastogi and Sinha, 2009; Silva-Stenico et al., 2011
*Anabaena vaginicola* and *Nostoc calcicola*	Phytohormones	Promote growth and development, enhances the production of ethylene that may be used as biofuel. Enhanced the production of defense enzymes and provide protection against different stresses.	Takahama et al., 2003; Tarakhovskaya et al., 2007; Hashtroudi et al., 2013
*Microcystis* sp., *Anabaena* sp., *Oscillatoria* sp., *Anabaenopsis* sp., *Nostoc* sp., *Hapalosiphon* sp., and *Lyngbya polychroa*	Toxins	Used for development of biocides that serve as antibiotics, anticancerous, and anti-inflammatory agents with relevant to pharmaceutical activities.	Burja et al., 2001; Biondi et al., 2004; Cardozo et al., 2007; Gunasekera et al., 2008

Organisms that are eukaryotic algae are designated with “^*^” mark.

### Phytoene/terpenoids

**Figure 3:**
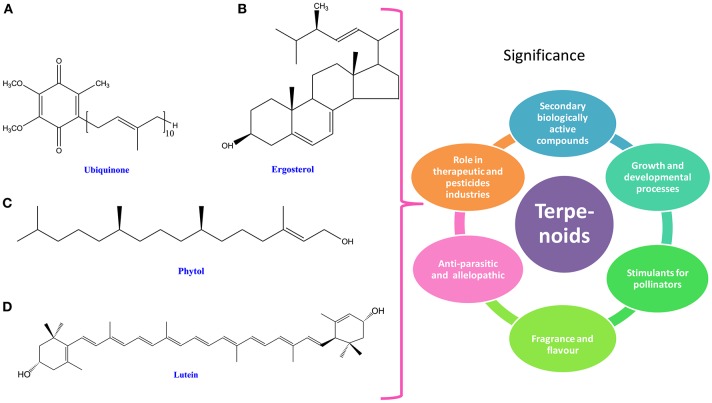
**Chemical structures and significance of terpenoid/ phytoene: (A)** ubiquinone, **(B)** ergosterol, **(C)** phytol, and **(D)** lutein obtained from different cyanobacteria and algae.

Terpenoids are the group of organic compounds widely distributed in cyanobacteria and algae (Keeling and Bohlmann, 2012). Depending upon their five-carbon isoprene unit, terpenoids have been classified into seven groups i.e., hemiterpenes (C5), monoterpenes (C10), sesquiterpenes (C15), diterpenes (C20), triterpenes (C30), tetraterpenes (C40), and polyterpenes (>C40; Keeling and Bohlmann, 2012; Singh and Sharma, 2015; Figure 3). Terpenoids not only play an essential role during preliminary growth and development processes but also stimulate attraction of pollinators (Gershenzon and Dudareva, 2007). These attributes make them more relevant as secondary biologically active compounds (Gershenzon and Dudareva, 2007). It has been reported that terpenes can be supplemented in products for their fragrances and flavors (Kirby and Keasling, 2009; Pattanaik and Lindberg, 2015). They are also emerging as advanced biofuel precursors like linear terpenes and being practiced to replace the biosynthetic diesel in the global market (Harvey et al., 2010; Pattanaik and Lindberg, 2015; Table 1). The function of pure monoterpenes has been suggested to be antiparasitic (Goulart et al., 2004; Bedoux et al., 2014). Several cyanobacterial species have allelopathic property which is attributed to the presence of significant amount of geranyl acetone that inhibits the growth of neighboring cyanobacterial species (Fischer, 1991). Terpenes are hydrocarbons synthesized within the cellular system, thus may be used as fuels. Furthermore, they can be used as a blend with the fossil gasoline in the spark ignition engine (Hellier et al., 2013). In recent years, terpenoids have gained more attention at commercial level due to their efficient roles in therapeutic and pesticide industries (de Carvalho and da Fonseca, 2006; Nichkova et al., 2009; Pattanaik and Lindberg, 2015; Table 1).

### Phytols

In cyanobacteria, phytols are crucial components of chlorophyll and also cofactors for the photosynthesis (Nowicka and Kruk, 2010; Kiyota et al., 2014). A wide range of terpenoids type compounds such as carotenoids and phytols are essential for chlorophyll, quinone prenyl tails, hormones and tocopherols that are biosynthesized through methylerythritol 4-phosphate (MEP) pathway in cyanobacteria and algae (Figure 3). During synthesis of carotenoids and phytols, a prerequisite precursor of terpenoids, geranylgeranyl pyrophosphate, comes out as a by-product through the MEP pathway, (Bentley et al., 2013; Pattanaik and Lindberg, 2015). In cyanobacteria, the native form of isoprenoids often consists of phytol of chlorophyll *a* and carotenoids and in complementary, isoprenoids also exist as cofactors of plastoquinone and phylloquinone (Kiyota et al., 2014). Studies have shown that limonene, which is a precursor for carotenoid and phytols' biosynthesis, is derived from the activity of limonene synthase enzyme. Under atmospheric pressure, limonene may be secreted from the cell without any specific treatment (Kiyota et al., 2014), which might help in enhancing its production. It has been reported that phytol may enhance an immunological response against tumor in a very beginning stage of carcinogenesis (Mukund et al., 2014). In addition, phytol may also serve as an anti-inflammatory agent (Shimizu and Tomoo, 1994; Silva-Stenico et al., 2014). Furthermore, it has been reported that phytol biosynthesis occurs in *Nitzschia ovalis* and *Phaeodactylum tricornutum* through the mevalonic acid pathway in which CO_2_ serves as a source of carbon (Cvejic and Rohmer, 2000; Fabris et al., 2014). In higher plants, isoprenoids, which are important for the photosynthetic machinery (phytol, plastoquinone, and carotenoids), are also produced by the MEP pathway (Kaspar, 1994; Paniagua-Michel et al., 2012; Pulido et al., 2012).

### Sterols

The key role of sterols is the regulation of membrane fluidity (Piironen et al., 2000; Volkman, 2003; Silvestro et al., 2013). Sterols are produced as by-products during isoprenoids biosynthesis. Until the discovery of sterols in 1968, it was considered that sterols are not produced in cyanobacteria. Reitz and Hamilton (1968), for the first time reported the presence of sitosterol and cholesterol in *Anacystis nidulans* and *Fremyella diplosiphon*. In the same year, De Souza and Nes (1968) reported the presence of seven unsaturated sterols in *Phormidium luridurn*. Cyanobacteria have been reported to produce both saturated and unsaturated sterols (Kohlhase and Pohl, 1988). The unsaturated sterols viz., cholesterol, chondrillasterol, stigmasterol, sitosterol, brassicasterol, campesterol, 22-dehydrocholesterol, isofucosterol, 24-ethyl-cholest-7-enol, 24-methyl-cholest-7-enol, 24-ethylcholesta-2,5-dienol, and 24-ethylcholesta-5,7, 22-trienol (Levin and Bloch, 1964; De Souza and Nes, 1968; Reitz and Hamilton, 1968; Nadal, 1971; Forin et al., 1972; Seckbach and Ikan, 1972; Teshima and Kanazawa, 1972; Paoletti et al., 1976; Perry et al., 1978; Figure 3), while some saturated sterols like 4α-methylsterols, 5α-cholestan-3β-ol, 24-methyl-5α-cholestan-3β-ol, and 24-ethyl-5α-cholestan-3β-ol have been reported from *Anabaena cylindrical, A. viguirei, A. solitaria, Nostoc carneum, Nodularia harveyana*, and *Microcystis aureguinosa* (De Souza and Nes, 1968; Patterson, 1971; Nishimura and Koyama, 1977). In a recent study on sterols content, Prochazkova et al. (2017) have reported the presence of sterols with a concentration up to 2.25 mg/L in water with cyanobacterial blooms.

Furthermore, in the biosynthesis of isoprenoids, wide ranges of cyclic and acyclic compounds are generated by the building block isopentenyl diphosphate leading to the formation of C30 oxygenated isoprenoid oxidosqualene. The biosynthesis of sterols takes place in the cytosol via series of chain reactions (Volkman, 2003; Fabris et al., 2014). In eukaryotic cells, sterols play essential role in various biochemical processes e.g., production of steroid hormones and also act as vital constituents of the cell membrane (Martin-Creuzburg and Von Elert, 2009). Moreover, from the nutritional point of view, sterols are good dietary sources for aqua-cultured organisms (Cardozo et al., 2007). The majority of sterols have planar structure with three β-hydroxy tetracycle containing a methyl- or ethyl-substituted hydrocarbon chain (C7-C11). They also exhibit C4, C14 methyl-substitution pattern in polycyclic form with varying degree and position of unsaturation (C5, C7, C8). The presence of fused ring system provides rigidity to sterol structure, which gives integrity as well as stability to the cell membrane and thus hold membranes together. Studies showed that the number of genes encoding enzymes such as D24-sterol C-methyltransferase, sterol-C-5-desaturase, or C-4 methyl sterol oxidase and sterol-C-methyltransferase actively participate in the biosynthesis of sterols in several cyanobacteria (Kaneko and Tabata, 1997; David Nes, 2011). Even though considerable progress has been made in identifying genes required for the biosynthesis of sterols, genetic evidence for the biosynthesis of sterols is still to be identified in cyanobacteria (Volkman, 2003).

### Free fatty acids

Among different kinds of metabolites, fatty acids are also very much important due to their key role in the metabolism. Cyanobacteria and algae contain some important fatty acids such as linolenic, linoleic, and arachidonic acids, which are prerequisite for healthy growth. Fatty acids and alcohols are the main ingredient of lipids and according to their configuration a great diversity in fats, phospholipids, glycolipids, and waxes may be found. In cyanobacteria, the structure of lipids may vary, based on the composition of vital fatty acids such as C18 linolenic and linoleic acids and their C20 derivative arachidonic and eicosapentaenoic acids (Singh et al., 2002). Several species of microalgae have capability of accumulating high amount of lipids, which could serve as good source of oil yield, as the average lipid content can vary between 1 and 70%, or even can reach upto 90% of dry weight (Mata et al., 2010).

Study showed that *Microcystis* cell lysate efficiently suppresses pumping of ions in gills of *Oreochromis mossambicus* due to the presence of fatty acids (Bury et al., 1998). In cyanobacteria, biosynthesis of fatty acids takes place through the action of an enzyme fatty acid synthase that utilizes acyl carrier proteins (ACPs; Froehlich et al., 1990; Kaczmarzyk and Fulda, 2010; Liu et al., 2011). In cyanobacteria, fatty acid synthesis (FAS) is carried out by a type II fatty acid synthase complex utilizing a freely dissociable acyl carrier protein (ACPs; an essential protein cofactor; Froehlich et al., 1990; Kaczmarzyk and Fulda, 2010; Liu et al., 2011). The products of FAS are released as acyl ACPs and may serve directly as substrates for acyltransferases thereby incorporating the fatty acids into membrane lipids (Frentzen et al., 1983; Kaczmarzyk and Fulda, 2010). From the biological activity point of view, fatty acids were reported to be anticarcinogenic, antibiotic, antifungal, and antiviral (Burja et al., 2001; El-Baz et al., 2013; Table 1). Among a wide variety of fatty acids, polyunsaturated fatty acids (PUFAs) are of great concern due to their health benefits and an increasing demand in the global market (Steinhoff et al., 2014). The presence of two or more double bonds (methylene-interrupted) in fatty acids (PUFAs) makes them more valuable from nutraceutical point of view. Further, these fatty acids also show biological activities in some medical practices, which make them more valuable in curing the obesity and cardiovascular diseases (Cardozo et al., 2007; Lee et al., 2016). Moreover, they are also involved in the regulation of various cellular processes such as transport of oxygen and electron, membrane fluidity, and heat adaptation (Funk, 2001; Cardozo et al., 2007).

Glycolipids (GLs) represent a complex carbohydrate made of sugar and fat by covalent bonds which have captured the growing interest of researchers. They are located in the chloroplast and thylakoid membranes and represent important signal and regulatory molecules (Siegenthaler and Murata, 1998; Hölzl and Dörmann, 2007; Harwood and Guschina, 2009; Boudière et al., 2014). The abundantly found glycolipids in microalgae are monogalactosyl diacylglycerols (MGDGs), digalactosyl diacylglycerols (DGDGs), and sulfoquinovosyl diacylglycerols (SQDGs), which are rich in PUFAs such as arachidonic (ARA, 20:4n-6), linoleic (LA, 18:2n-6), α-linolenic (ALA, 18:3n-3), docosahexaenoic (DHA, 22:6n-3), and eicosapentaenoic (EPA, 20:5n-3) fatty acids (Harwood and Guschina, 2009; He et al., 2011; Kim et al., 2013; da Costa et al., 2016). SQDG is a negatively charged GL having a monoglycosyl diacylglycerol with a sulfonic acid present in the 6th position of monosaccharide moiety [1,2-diacyl-3-*O*-(6-sulfo-6-deoxy-α-D-glucosyl)-*sn*-glycerol] (Reshef et al., 1997; Naumann et al., 2007). SQDGs participate in signaling and in the coordination between chloroplast lipids and cytosolic partners. MGDG, DGDG, and SQDG are chief components of the chloroplast lipids (Siegenthaler and Murata, 1998; Wang and Benning, 2012; Boudière et al., 2014). MGDG represents about 20% outer and 40–55% of the inner envelope of chloroplast and thylakoid membranes (Siegenthaler and Murata, 1998). DGDG consists of about 15–35% and SQDG about 2–40% of total lipids in the chloroplast and thylakoid membranes (Siegenthaler and Murata, 1998). SQDGs content in microalgae is comparatively high in comparison with *Arabidopsis thaliana* (2–10%; Siegenthaler and Murata, 1998; Muhlroth et al., 2013). GLs are important antitumor agents. SQDGs cause inhibitory effects on tumor cell growth and are a potent inhibitor of DNA polymerase that may result into the death of tumor cells, especially under active proliferation conditions (Hossain et al., 2005; Guschina and Harwood, 2006; Chirasuwan et al., 2007). A type of sulfate-group containing glyceroglycolipid was separated from the cyanobacterium *L. lagerheimii* (Gustafson et al., 1989) that is able to inhibit the replication of HIV. The antiviral properties of nGLs were confirmed on SQDG isolated from *Spirulina platensis* and *Porphyridium purpureum*. The sulfonate group may be responsible to carry out the antiviral activity of SQDGs (Plouguerné et al., 2014). It was suggested that lipophilic groups on SQDG interact with the positive charged side of DNA polymerase.

### Photoprotective compounds

**Figure 4:**
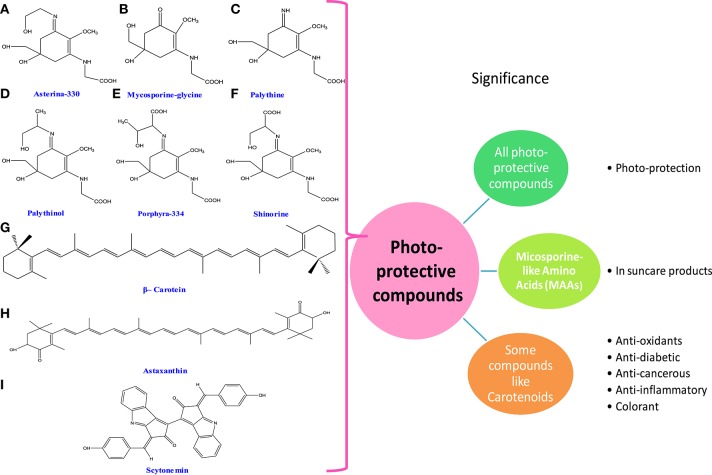
**Chemical structures and significance of photoprotective compounds: (A)** asterina-330, **(B)** mycosporine-glycine, **(C)** palythine, **(D)** palythinol, **(E)** porphyra-334, **(F)** shinorine, **(G)** β-carotene, **(H)** astaxanthin, and **(I)** scytonemin obtained from different cyanobacteria and algae.

Ultraviolet radiation (UVR) causes a wide range of harmful biological effects on living system. In cyanobacteria and algae, a number of biologically active compounds such as carotenoids, mycosporine-like amino acids (MAAs), and scytonemin have been isolated (Figure 4). They exhibit photoprotective properties under radiation stress. The biosynthesis of these compounds may be affected by different environmental stimuli including the fluctuation of light intensity, different wavelengths of UV radiation, nutrient limitation, and several other stresses (Rastogi et al., 2010). The MAAs are intracellular, colorless, small, and hydrophilic compounds. They have a great potential to dissipate excess energy in the form of heat thereby avoiding the formation of toxic oxygen radicals (Conde et al., 2000; Groniger and Hader, 2000; Whitehead and Hedges, 2005; Oren and Gunde-Cimerman, 2007). Moreover, it has been reported that MAAs not only play protective role under radiation stress but they could protect primary and secondary consumers if consumed by them (Helbling et al., 2002; Bhatia et al., 2011; Table 1).

#### Mycosporine-like amino acids (MAAs)

Mycosporine-like amino acids (MAAs) are a family of intracellular compounds engaged in the protection of aquatic organisms against solar radiation. Structurally, in their formation nitrogen substituent of amino acids and cyclohexenimine or cyclohexenone chromophore conjugated with one or two amino acids is involved, having absorption maxima ranging from 310 to 362 nm (Singh et al., 2008; Bhatia et al., 2011). MAAs are produced in several cyanobacteria, rhodophyta, and several other groups of microalgae (Sinha et al., 2007; Carreto and Carignan, 2011; Rastogi et al., 2015; Rastogi and Madamwar, 2016). They have absorption maxima in UV range (Takano et al., 1979; Bhatia et al., 2011; Kannaujiya et al., 2014). Studies have shown that MAAs originate from shikimate pathway, but the exact route of their biosynthesis is still unknown. Favre-Bonvin et al. (1987) revealed that the precursor of the six-membered carbon MAAs i.e., 3-dehydroquinate (an intermediate of the shikimate pathway) has been commercially explored for protection of skin as suncare products and other non-biological materials such as photostabilising additives in varnish, paint, and plastics (Bandaranayake, 1998). It is thought that cyanobacteria and algae are the ancestors of MAAs (Nakamura et al., 1982; Klisch and Häder, 2008), which evoke protective responses. The high molar coefficients (ε = 28,100–50,000 M^−1^ cm^−1^) as well as UV-absorption maxima ranging from 310 and 362 nm along with the photostability in both fresh and sea water in presence of photosensitizers and resistant to specifically abiotic stressors such as pH, temperature, various solvents, and UV radiation (UVR) provide strong evidence in favor of MAAs as photoprotective compounds (Whitehead and Hedges, 2005; Yoshiki et al., 2009; Shahidi and Zhong, 2010; La Barre et al., 2014). The protection against UV-B damage provided by MAAs mainly depends on the species and the pigments localization within. Significant, but limited, protection has been reported for various cyanobacteria with MAAs located in the cytoplasm. In case when MAAs located in the cytoplasm, according to Garcia-Pichel and Castenholz (1993) only 10–26% of the photons are absorbed by the pigment. MAAs are thought to play an important role in photoprotection as the MAAs are located in the extracellular glycan in *Nostoc commune*. According to Böhm et al. (1995), pigments present in cell membranes or target absorbs two out of three photons reaches within the cell. In *N. Commune*, two UVA/B-absorbing pigments with absorption maxima at 312 and 335 nm were reported to be present in colonies when exposed to high solar radiation (Scherer et al., 1988; Ferroni et al., 2010). Out of them, one was the first mycosporine covalently linked to the oligosaccharides and was reported to be located in the extracellular glycan (Hill et al., 1994; Böhm et al., 1995). These compounds are enough capable of effectively dissipating absorbed radiation in the form of heat without producing reactive oxygen species (ROS; Conde et al., 2000). It has also been reported that MAAs provide protection from UVR not only in their producers but also to primary and secondary consumers via food chain (Helbling et al., 2002). Carreto et al. (1990) have shown that after application of 3-(3,4-dichlorophenyl)-1,1-dimethylurea (DCMU), the biosynthesis of MAAs was inhibited in the alga *Alexandrium excavatum*, which suggests a close relationship between the photosynthetic process and MAAs synthesis. The biosynthetic reactions of MAAs take place in two steps, the first step involves the reduction of carboxylic group of 3-dehydroquinate and the second step involves methylation of the hydroxyl group at C4 and the attachment of one (mycosporine-glycine) or two amino acids or amino alcohols (Klisch and Häder, 2008).

#### Scytonemin

Scytonemin (MW 544 Da), a photo-protective compound is a dimer of indolic and phenolic subunits. It was firstly reported in some terrestrial cyanobacterial sp. as a yellowish-brown lipid soluble pigment located in the exopolysaccharide sheath (Garcia-Pichel and Castenholz, 1991; Rothrock and Garcia-Pichel, 2005; Wada et al., 2013; Rastogi et al., 2015). Although, scytonemin is predominantly found in green oxidized form, it has two more forms viz., reduced (fuscorhodin; red in color) and oxidized (fuscochlorin; yellow in color; Garcia-Pichel and Castenholz, 1991; Wada et al., 2013). Recently from the organic extracts of *Scytonema* sp., dimethoxyscytonemin, tetramethoxyscytonemin, and scytonin pigments have been isolated (Bultel-Poncé et al., 2004; Grant and Louda, 2013; Rastogi et al., 2014). The *in vivo* absorption maxima of scytonemin is at 370 nm while purified scytonemin has absorption maximum at 386 nm, but it also absorbs significantly at 252, 278, and 300 nm that's why it probably helps cyanobacteria to survive under lethal UV radiation. Studies have shown that scytonemin alone is sufficient to reduce the risk of damage caused by the most lethal UV-C radiation (Dillon and Castenholz, 1999; Rastogi et al., 2013). Scytonemin can effectively reduce photosynthesis inhibition by UV-A radiation and also can reduce photobleaching of chlorophyll *a* (Cockell and Knowland, 1999; Gao and Garcia-Pichel, 2011). The role of scytonemin as an UV-sunscreen has been confirmed in the terrestrial cyanobacterium *Chlorogloeopsis* sp. (Garcia-Pichel et al., 1992; Portwich and Garcia-Pichel, 2003). Scytonemin is highly stable in response to different stressors such as strong UV radiation, temperature, etc., and carry out its screening activity without any additional metabolic investment even after prolonged physiological inactivity when other ultraviolet protective mechanisms like active repair of damaged cellular components would be ineffective (Brenowitz and Castenholz, 1997). In addition, due to the higher screening potential of scytonemin, it may be used as a sunscreen in cosmetics for human beings (Rastogi et al., 2010, 2015; Table 1).

#### Carotenoids

A wide occurrence of carotenoid pigments is an essential phenomenon in the microorganism, animal, and plant life. Carotenoids, the accessory pigments in photosynthesis are polymers of isoprene units containing 40 carbons and up to 15 double bonds arranged in a conjugated manner (Bramley and Mackenzie, 1988; Solomons and Bulux, 1994; Yuan et al., 2015). Compounds consisting of hydrocarbons are only the carotenes, while those having oxo, hydroxyl, or epoxy groups fall under the category of xanthophyll. The number and positions of these double bonds identify the spectral properties of carotenoids, which typically absorb the light in range of 400 and 500 nm. Among different forms of carotenoids, two major forms i.e., β-carotene and echinenone are of great importance. In addition, several others forms such as astaxanthin, β-cryptoxanthin, zeaxanthin, canthaxanthin, and 30-hydroxyechinenone have a great significance in cyanobacteria and algae (Mochimaru et al., 2005; Shah et al., 2016). They play multifunctional roles such as colorant, precursors of visual pigments, as well as contribute to improve the antioxidant status in plants and algae. The most dynamic form of carotenoids, β-carotene, and its derivative compounds also may act as the precursor for retinoic acid, retinal, and vitamin A and thus improving the nutritional value, vision, and cellular differentiation in mammals (Olson, 1993; Seino et al., 2008; Table 1). Algal β-carotene provides protection against atherosclerosis in mouse and humans (Munawer and Mazharuddin, 2011). It has been reported that in diabetic patients, β-carotene rich algae *Dunaliella* sp. has the potentiality of controlling cholesterol, plasma triglycerides level, and also delays development of atherosclerosis by inhibiting oxidation of low density lipoprotein (LDL) and high density lipoprotein (HDL; Sanchez and Demain, 2008; Munawer and Mazharuddin, 2011). Astaxanthin, a keto-carotenoid pigment obtained from the green alga *Haematococcus pluvialis* is of commercial application. Astaxanthin accumulates under unfavorable condition, when thin-walled flagellated stage of the algae changes into red thick-wall resting stage and it may contribute up to 4–5% of dry weight (Froehlich et al., 1990; Ambati et al., 2014). Astaxanthin usually serves as food additive for salmon, trout, and shrimp for many aquacultures and also for the poultry industry and food coloring agent (Frentzen et al., 1983; Higuera-Ciapara et al., 2006; Ambati et al., 2014). Because of its strong antioxidant activity, astaxanthin is consumed as neutraceuticals in the form of encapsulated product and *Haematococcus* (*H. pluvialis*, a green alga) rich in astaxanthin is being sold in market as dietary supplement for human being (Frentzen et al., 1983; Guerin et al., 2003; Bishop and Zubeck, 2012). Sayanova and Napier (2004) have reported that astaxanthin can be effective against several diseases like cancer, diabetes, diabetic nephropathy, inflammatory diseases, as well as for syndromes like metabolic syndrome and neurodegenerative diseases.

### Polysaccharides

**Figure 5:**
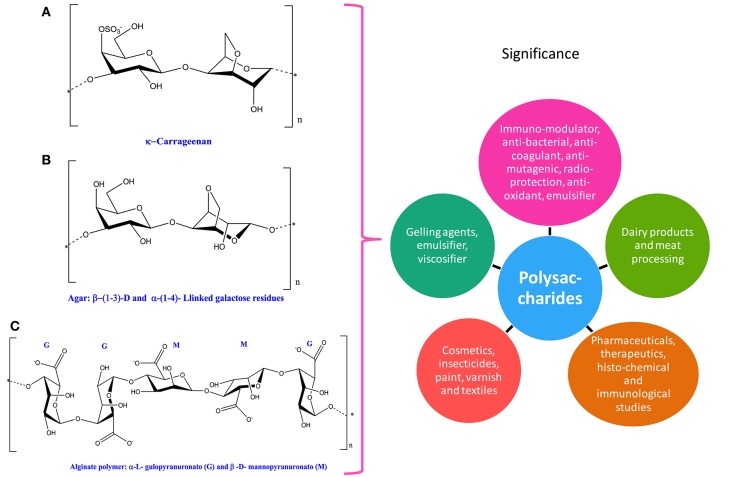
**Chemical structures and significance of phycocolloid compound: (A)** carrageenan, **(B)** agar, and **(C)** alginate polymer obtained from different cyanobacteria and algae.

All organisms possess biochemical structures having linearly attached 40–50 different monosaccharaides (hexoses and pentoses) connected by glycosidic linkage along with some other substituent like acyl, amino acids, or sulfates (Figure 5). These polysaccharides serve as the source of carbon and energy and are excreted during normal as well as stressful physiological processes. They are being utilized as thickening or gelling agents (Delattre et al., 2009, 2011; Kraan, 2012). In addition, they have immunomodulatory, antibacterial, anticoagulant, antimutagenic, radioprotective, anti-oxidative, antiulcer, anticancer, and anti-inflammatory properties (Kraan, 2012; Misurcova et al., 2015; de Jesus Raposo et al., 2015). Cyanobacteria and algae have been suggested to synthesize polysaccharides and the polysaccharides produced by microalgae ranges from ~0.5 g/L up to 20 g/L (Markou and Nerantzis, 2013). The process how these polysaccharides are produced and extracted from microalga and cyanobacteria has been reviewed by Delattre et al. (2016). Different types of polysaccharides are obtained from both cyanobacteria and algae; some of them are discussed in the following sections.

#### Phycocolloids

Phycocolloids are unique type of polysaccharides synthesized by various species of seaweeds. Among different types of phycocolloids, carrageenan, agar, and alginates are of great importance due to their multifunctional uses (Figure 5 and Table 1). The significant roles of polysaccharides as antioxidants, antiviral, antitumoral, and anticoagulant have been well-documented in cyanobacteria and algae (Smit, 2004; Kılınç et al., 2013; Minicante et al., 2016). Agar and carrageenan, the sulfated polysaccharides are extracted from red algae, while alginates, that are binary polyurodine consisting of guluronic and mannuronic acids have been isolated from brown algae (Smit, 2004; Kılınç et al., 2013). Multifunctional uses of phycocolloids as emulsifier, viscosifier, and gelling agent are attractive for scientists and industry (Cardozo et al., 2007).

#### Carrageenan

Carrageenans are high molecular weight compounds that are formed through the replicating disaccharide units with modifications in 4-linked α-galactopyranose and/or 3,6-anhydro-D-galactopyranose and 3-linked β-D-galactopyranose also known as sulfated _D_-galactans (Jiao et al., 2011). Depending upon the existence of sulfate groups such as 3,6-anhydrogalactose on the 4-linked residue and their amount and allocation, they can further form a diverse range of carrageenans (Pereira et al., 2009; Blanco-Pascual et al., 2014). For instance, λ-carrageenan consists of three sulfate groups per disaccharide unit where the third sulfate group is present at the C6 position of the 4-linked residue, but these 4-linked residues lack 3,6-anhydride bridge (Jiao et al., 2011). In nature, λ-carrageenan is produced by red algae *Chondrus* and *Gigartina* (Zhou et al., 2006). The viscous property of carrageenan makes it more valuable in dairy industry, meat processing, and other miscellaneous products like toothpaste, air freshener gels, and pet food (Table 1).

#### Agar

Agar may be isolated by the boiling of certain species of algae, which results into the breaking of the cell wall and release of two structural polysaccharides. Furthermore, the binding of these two polysaccharides results in agar formation, which is dried in the oven and grounded into a fine powder that is ideal for storage (Cardozo et al., 2007). Seaweed galactans, which are collectively known as agar, contains α (1 → 4)-3,6-anhydro-_L_-galactose and β(1 → 3)-D-galactose (Cardozo et al., 2007). Despite the fact that the biosynthetic pathway of agar is well-known (Hammingson et al., 1996; Siow et al., 2013), the processes implicated in converting precursors i.e., mannose and glucose into an agar via _D_-and _L_-galactose are poorly understood (Goncalves et al., 2002; Siow et al., 2013). In food industries, agar is commonly used as emulsifying, stabilizing, and thickening agent. It is also used as a mild laxative component in pharmaceutical products. Moreover, in microbiology agar may serve as a growth medium for bacteria and fungi in Petri dishes due to its solidifying nature, which is ideal for experiments incubated at human body temperature.

#### Alginate

Alginate, also called alginic acid or algin, is widely found in cyanobacteria and algae and mainly made up of linear polysaccharides, which contain α-_L_-guluronic acid and 1,4-linked β-_D_-annuronic (Cardozo et al., 2007). Alginates are derivatives of alginic acid extracted from brown algae such as *Laminaria* and are extensively used in cosmetics, pharmaceuticals, insecticides, paints, and printers' ink (Raja et al., 2013). Moreover, in the textile industry, for sizing the cotton yarns alginate is being used as a gelling agent. Brown algae, being the good source of alginates, are very popular in several food and pharmaceutical industries (Raja et al., 2013).

#### Lectins

Among extensive range of biologically active compounds, it is necessary to emphasize on lectins. Lectins or agglutinins are synthesized in cyanobacteria and algae and are the complex form of proteins, having the ability to bind directly with carbohydrates without changing the property of carbohydrate to which they bound (Lam and Ng, 2011). Although, lectins adopt the ordinary process of binding, the significance of sugar may not be the same (Lam and Ng, 2011). Their specificity of carbohydrates binding makes them valuable candidates for application in histo-chemical and immunological studies and also in identifying sugar type on the cell surface. In biological sciences particularly in medicine, lectins are valuable for identification of diseases pertaining to the modification in the synthesis of glycan, such as the typing of blood group on the basis of the secretor status and malignancy (Rudiger and Gabius, 2001; Kumar et al., 2012). Lectins are commonly used as therapeutic agents because they have unique ability of binding epithelium of intestine and enhance diffusion of drugs (Chowdary and Rao, 2004). Keeping their therapeutic importance into consideration, numerous lectins, i.e., scytovirin, microvirin, agglutinin, and cyanovirin-N have been isolated from several cyanobacteria such as *Scytonema varium, Mycrocystis* sp., *Nostoc ellipsosporum*, and *Oscillatoria agardhii* (Bewley et al., 1998; McFeeters et al., 2007; Ziemert et al., 2010; Mandal and Rath, 2014). Furthermore, lectins are used in anticipating transmission of HIV due to the interaction of glycans with HIV gp120 and thus, exhibit a great potential for antiviral activities (Bewley et al., 2004; Huskens et al., 2010).

### Halogenated compounds

**Figure 6:**
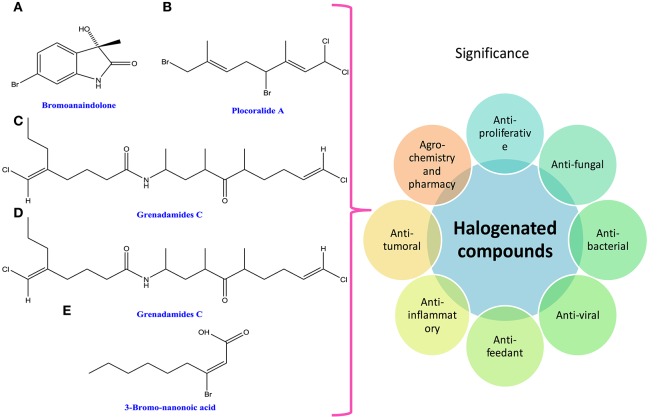
**Chemical structures and significance of halogenated compound: (A)** bromoanaindolone, **(B)** plocoralide A, **(C)** grenadamides B, **(D)** grenadamides C, and **(E)** 3-bromo-nanonoic acid polymer obtained from different cyanobacteria and algae.

Halogenated compounds have been isolated mainly from phaeophyceae and rhodophyceae, dispelling the general rumors that they are only man-made. The wide occurrence of halogenated compounds in cyanobacteria and algae may be characterized as acetogenins, phenols, terpenes, indoles, fatty acids, and volatile halogenated compounds (i.e., dibromomethane, chloroform, and bromoform; Butler and Carter-Franklin, 2004; Figure 6). They are very important from the pharmacological point of view as they show biological activities like antiproliferative, antifungal, antibacterial, antiviral, antifeedant, antifouling, anti-inflammatory, cytotoxic, ichthyotoxic, insecticidal, and antitumoral (Vairappan et al., 2001; Cabrita et al., 2010; Table 1). A lot of biologically active peptides, aeruginosin, and cyanopeptolin, which are the protease inhibitors, have been well-recognized in several cyanobacteria and have great role in agrochemistry and pharmacy (Silva-Stenico et al., 2011). Moreover, the diverse ranges of halogenated alkanes such as CH_3_Cl, CH_3_Br, CH_3_I, CH_2_Br_2_, and CHBr_3_ are produced by the brown alga *Macrocycstis pyrifeara* (Manley et al., 1992; Dembitsky and Tolstikov, 2003). Similarly, different genera of brown algae such as *Eisenia arborea, Egregia menziesii, Custoseria osmundacea, Laminaria farlowii*, and *Prochlorococcus marinus* also produce CH_3_I, CHBr_3_, and CH_2_Br_2_ (Manley et al., 1992; Dembitsky and Tolstikov, 2003; Hughes et al., 2011).

### Phytohormones

It has been established that different genera of cyanobacteria and algae significantly accumulate and release a diverse group of phytohormones including auxins, gibberellins (GA), cytokinins (CKs), and ethylene (ET) that are involved in plants' growth and development (Stirk et al., 2002; Hussain and Hasnain, 2011; Gayathri et al., 2015). Phytohormones such as indole-3-acetic acid (IAA) and CKs, excreted by symbiotic cyanobacteria are consumed by the host plants during their growth and developmental processes (Hussain and Hasnain, 2010). Furthermore, the study by Hussain et al. (2013) showed that endophytic *Nostoc* strains have the capability to produce equal amount of phytohormones (IAA and CKs) in root cells of both rice and wheat and in the same study they showed that *ipt* gene is mostly activated during the production of CKs and IAA in *Nostoc* sp. After knocking out this gene, a significant decrease in CKs and IAA accumulation was noticed. The basic reason of the deactivation of the *ipt* gene is homologous recombination in the background of *Nostoc* sp., after that, the synthesis of zeatin was drastically reduced followed by a significant decrease in the growth of the mutant strain. Numerous plant growth regulators (PGRs) such as amino acids, sugars, vitamins that may up-regulate the growth of the vascular plant, have been isolated from different genera of cyanobacteria and algae (Misra and Kaushik, 1989; Karthikeyan et al., 2007; Table 2). It is evident that among different phytohormones only two i.e., auxins and CKs perform their action at very low concentrations. Moreover, Stirk et al. (2002) and Stirk et al. (2009) also reported that *Cyanophyta* and *Chlorophyta* exhibited CK-like activity, which could be beneficial to agricultural crops. Moreover, algae and cyanobacteria are the great sources of numerous oxylipins, including jasmonic acid and its volatile methyl ester. The presence of these hormones has been observed in various microautotrophs such as green algae (*Dunaliella tertiolecta, Dunaliella salina*, and *Chlorella* sp.), euglenophyta (*Euglena gracilis*), red alga (*Gelidium latifolium*), and cyanobacteria (*Spirulina* sp.; Karthikeyan et al., 2007). In brown algae *Ectocarpus siliculosus*, IAA has been shown to play the regulatory role in the induction of signaling pathway and also in relaying cell-cell positional information (Le Bail et al., 2010). In another study, it was noticed that cleavage of specific cyclic epoxy-xanthophylls may have a crucial role in initiating the formation of abscisic acid (ABA; Rock and Zeevaart, 1991). In cyanobacteria, ABA functions under salt stress condition while in other class of algae it functions as a stress molecule under drought, nutrient, osmotic, oxidative, and salt stresses (Kobayashi et al., 1997; Yoshida et al., 2003, 2004; Lu et al., 2014). In the alga, *Nannochloropsis oceanic*, under nitrogen deprivation, the biosynthetic pathway of CKs and ABA was found to be transcriptionally down-regulated and up-regulated, respectively. Recently, it was found that ethylene (ET), produced by a green alga *Spirogyra pratensis* regulates the cell development process, revealing that ET has emerged before land colonization (Ju et al., 2015). In two green algae, *Chlamydomonas* sp. and *Chlorella* sp. both Gibberellic acid (GA) GAs and ET have been found to be involved in growth, senescence and different biological activities (Yordanova et al., 2010; Park et al., 2013; Tate et al., 2013). These phytohormones, obtained from the diverse algae and cyanobacteria, may be applied commercially in agricultural land to enhance the crop productivity (Table 2).

**Table 2:** **An overview of various phytohormones produced by algae and their potential implication in microbial biotechnology**.

**Phytohormones**	**Cyanobacteria/algae**	**Relevant findings in microalgae**	**Potential implication for microalgal biotechnology**	**References**
Abscisic acid (ABA)	Anabaena variabili, Ascophyllum nodosum^*^, Chlamydomonas reinhardtii^*^, Chlorella minutissima^*^, Chondracanthus^*^ sp., Coscinodiscus granii^*^, Draparnaldia mutabilis^*^, Dunaliella^*^ sp., Gelidium^*^ sp., Gracilaria^*^ sp., Gracilariopsis^*^ sp., Hypnea^*^ sp., Nannochloropsis oceanic^*^, Nostoc muscorum, Porphyra^*^ sp., *Trichormus variabilis, Synechococcus leopoliensis*	Exogenous ABA decreases growth rate in Nannochloropsis oceanica^*^ and Coscinodiscus granii^*^ Exogenous ABA improves stress tolerance to dehydration in Haematococcus pluvialis^*^, higher salinity in *Dunaliella* sp.,^*^ and Chlamydomonas reinhardtii^*^; nitrogen deprivation in Nannochloropsis oceanica^*^; osmotic stress in Chlamydomonas reinhardtii^*^	Improvement in stress tolerance.	Boyer and Dougherty, 1988; Hirsch et al., 1989; Kentzer and Mazur, 1991; Zahradnıckova et al., 1991; Marsšálek et al., 1992; Tominaga et al., 1993; Kobayashi et al., 1997; Yoshida et al., 2003; Hartung, 2010; Yokoya et al., 2010; Lu et al., 2014; Stirk et al., 2014
Auxins	*Anabaena* sp., Chlorella minutissima^*^, Chlorella pyrenoidosa^*^, Chondracanthus^*^ sp., *Chroococcidiopsis* sp., Ectocarpus siliculosus^*^, Gelidium^*^ sp., Gracilaria^*^ sp., Gracilariopsis^*^ sp., Hypnea^*^ sp., *Nostoc* sp., *Oscillatoria* sp., *Phormidium* sp., Porphyra^*^ sp., Prionitis lanceolate^*^, Scenedesmus armatus^*^, *Synechocystis* sp.	Exogenous indole-3-acetic acid (IAA) improves growth rate in Chlamydomonas reinhardtii^*^, Chlorella sorokiniana^*^, Chlorella vulgaris^*^, Haematococcus pluvialis^*^, *Nostoc* sp., Phaeodactylum tricornutum^*^, and Pleurochrysis carterae^*^, and oil content in Chlamydomonas reinhardtii^*^, Haematococcus pluvialis^*^, and Phaeodactylum ricornutum^*^	Elevation of microalgal growth rate, biomass production, oil content, and stress tolerance.	Ashen et al., 1999; Mazur et al., 2001; Sergeeva et al., 2002; Le Bail et al., 2010; Hussain et al., 2010; Maor, 2010; Yokoya et al., 2010; Mazhar et al., 2013; Park et al., 2013; Piotrowska-Niczyporuk and Bajguz, 2014; Stirk et al., 2014
Cytokinins (CK)	*Anabaena* sp., *Calothrix* sp., Chlorella minutissima^*^, *Chlorogloeopsis* sp., Chondracanthus^*^ sp., *Chroococcidiopsis* sp., Ecklonia^*^ sp., Ecklonia maxima^*^, Gelidiums^*^ sp., Gigartina clathrate^*^, Gracilaria^*^ sp., Gracilariopsis^*^ sp., Hypnea^*^ sp., Laminaria pallid^*^, Nannochloropsis oceanic^*^, *Oscillatoria* sp., *Phormidium* sp., Porphyra^*^ sp., Rhodospirillum^*^ sp., *Synechocystis* sp.	Exogenous CK improves cell cycle progression in Nannochloropsis oceanica^*^; growth rate in Chlamydomonas reinhardtii^*^, Nannochloropsis oceanica^*^, and oil content in Chlamydomonas reinhardtii^*^, Haematococcus pluvialis^*^, and Phaeodactylum tricornutum^*^ Elevated temperature led to increase in CK contents in Ecklonia maxima^*^ and Macrocystis pyrifera^*^	Elevation of microalgal growth rate, oil content, and stress tolerance.	Jennings, 1969; Tian et al., 2006; Tsavkelova et al., 2006; Hussain et al., 2010; Stirk et al., 2011; Park et al., 2013; Lu et al., 2014; Stirk et al., 2013, 2014
Ethylene (ET)	*Anabaena* sp., *Calothrix* sp., Chlorella pyrenoidosa^*^, *Cylindrospermum* sp., Ecklonia maxima^*^, *Nostoc* sp., Padina arborescent^*^ sp., Porphyra tenera^*^, *Scytonema* sp., *Synechococcus* sp.	ET take part in programmed cell death of microalgae in Chlamydomonas reinhardtii^*^	Enhancement of microalgal growth rate as well as biomass productivity.	Watanabe and Kondo, 1976; Kreslavsky et al., 1997; Tsavkelova et al., 2006; Yordanova et al., 2010
Gibberellins (GA)	*Anabaenopsis* sp., Chlamydomonas reinhardtii^*^, Chlorella^*^ sp., *Cylindrospermum* sp., Ecklonia radiate^*^, Hypnea musciformis^*^, Nannochloropsis oceanic^*^, *Phormidium foveolarum*	Exogenous GA stimulates astaxanthin biosynthesis in Haematococcus pluvialis^*^ Exogenous GA improves growth rate in Chlamydomonas reinhardtii^*^	Increased algal growth rate and biomass productivity. Target chemical production.	Jennings, 1968; Gupta and Agarwal, 1973; Tsavkelova et al., 2006; Park et al., 2013; Stirk et al., 2013; Voß et al., 2014

Organisms that are eukaryotic algae are designated with “^*^” mark.

### Cyanotoxins

**Figure 7:**
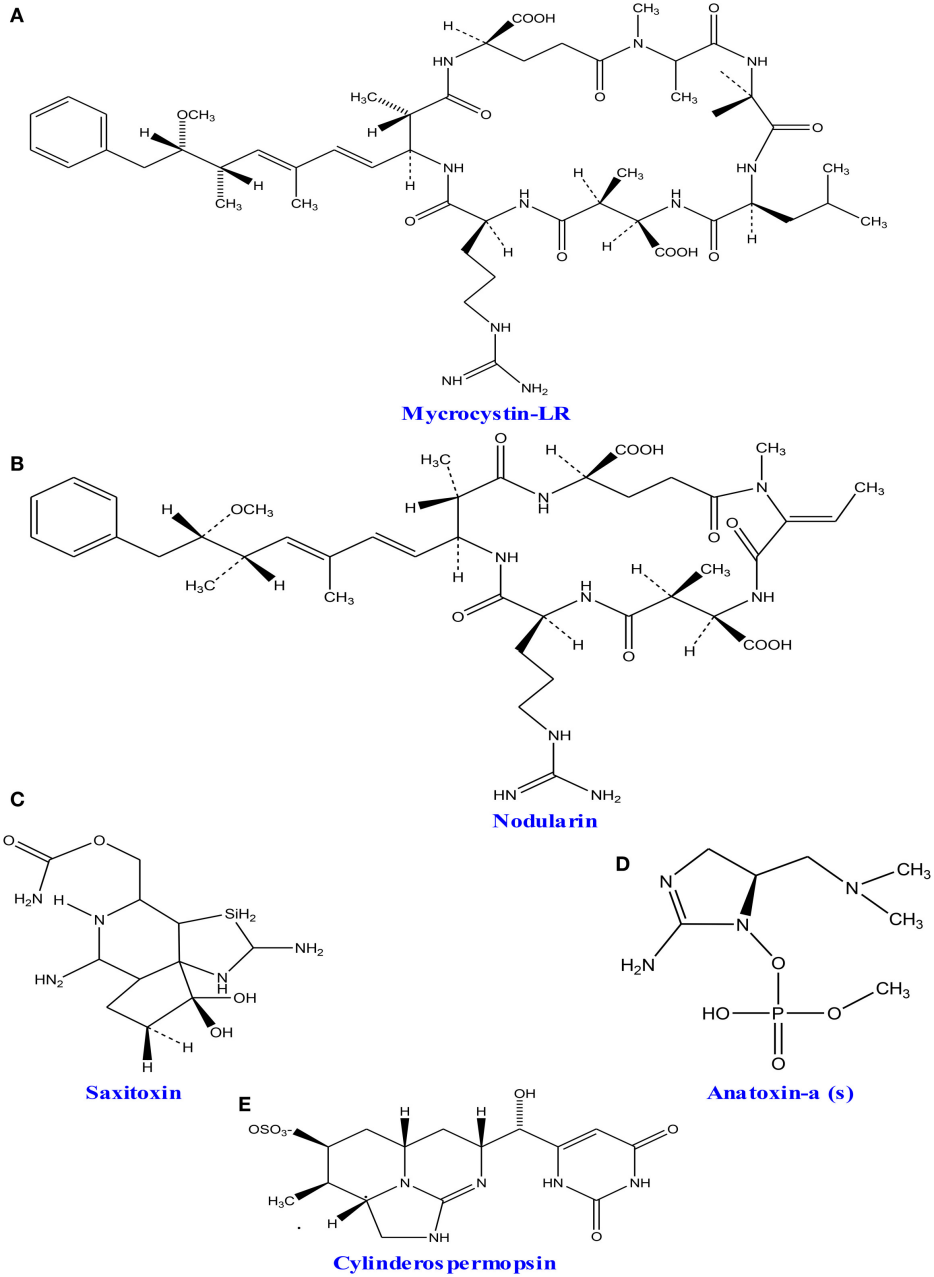
**Chemical structures of cyanotoxins: (A)** microcystin-LR, **(B)** nodularin, **(C)** saxitoxin **(D)** anatoxin- a, and **(E)** cylinderosprmopsin obtained from different cyanobacteria.

A wide group of toxins, which are a part of secondary metabolites, are secreted by different marine as well as fresh water algae and cyanobacteria. The excess level of nutrients like nitrogen and phosphorus creates algal blooms, which result into serious problems of water quality by producing different form of toxins (Codd et al., 2005). Studies showed that five active groups of toxins including neurotoxins (anatoxins and saxitoxins), cytotoxins (cylindrospermopsin), hepatotoxins (nodularin and microcystins) dermatotoxins and irritant toxins or endotoxins (lypopolysaccharides and lipopolysaccharides) produced by cyanobacteria and algae are of great concern due to their serious impact on human health (Wiegand and Pflugmacher, 2005; Gacsi et al., 2009; Figure 7 and Table 3). Toxins released from freshwater and marine algae can accumulate upto some extent in several aquatic organisms especially in fish, mollusks and seafood (Landsberg, 2002; Cazenave et al., 2005). Bioaccumulation of these compounds can severely affect health of domestic animals, humans and wildlife thereby causing several toxicological effects like toxicity within the cell (cytotoxic), skin (dermatotoxicity), hepatotoxicity, and neurotoxicity (Kujbida et al., 2006). The most common freshwater algal toxins such as anatoxin-a, cylindrospermopsin, microcystins, and saxitoxins are obtained from cyanobacterial strains viz., *Anabaena, Microcystis, Nostoc*, and *Oscillatoria* sp. (Codd et al., 2005). Cylindrospermopsin is another toxin which is an alkaloid produced (with strain-specific production; Valerio et al., 2005) in the cyanobacterial strains of *Aphanizomenono valisporum* (in Australia and Israel), *Cylindrospermopsis raciborskii* (in Australia, Hungary, and the United States), *Umezakia natans* (in Japan), and *Anabaena* sp. (Torokne et al., 2004; Neumann et al., 2007). Neurotoxins have been classified into three main classes (i) anatoxin-a, the first powerful cyanotoxin (Koskinen and Rapoport, 1985), (ii) saxitoxin from *Anabaena circinalis* (a cyanobacterium) in Australia and *Aphanizomenon flosaquae* (a cyanobacterium) in North America (Mahmood and Carmichael, 1986; Fergusson and Saint, 2000; Al-Tebrineh et al., 2010) that cause widespread animal mortality; and (iii) anatoxin-a(s) that acts as a potent irreversible acetyl cholinesterase inhibitor (Devic et al., 2002).

**Table 3:** **An overview of cyanotoxins produced by algae and cyanobacteria and their potential impacts on other organisms**.

**Cyanotoxins**	**Source**	**Nature**	**Effect**	**References**
Cylindrosper-mopsins (Hepatotoxic, nephrotoxic, and cytotoxic)	*Anabaena, Aphanizomenon ovalisporum, Umezakia natan, Raphidiopsis curvata*, and *Cylindrospermopsis raciborskii*	Guanidine alkaloid	Inhibitor of protein biosynthesis, glutathione synthesis, implicate cytochrome P-450, overexpression of DNA damage repair proteins genotoxic.	Humpage et al., 2000; Neumann et al., 2007
Dermatotoxins Aplysiatoxins	Lyngbya, Schizothrix, Gracilaria coronopifolia^*^, and *Oscillatoria*	Alkaloid (phenolic bislactone)	Inflammatory agents, protein kinase C activators. Dilation of the lymphatic vessel and congestion of capillaries, diarrhea, and Fibrin deposition in the dilated pulmonary artery followed by bleeding.	Fujiki et al., 1982; Wiegand and Pflugmacher, 2005
Lyngbyatoxin	*Lyngbya majuscula, Oscillatoria*, and *Schizothrix*	Alkaloid	Causes erythema (dermatitis), blisters, and necrosis in mammals; potent tumor promoters.	Fujiki et al., 1982; Taylor et al., 2014
Endotoxins Lipopolysacc-haride	All cyanobacteria	Lipopolysaccharide	Inflammatory agent, gastrointestinal irritants.	Stewart et al., 2006
Hepatotoxins Heptapeptide	*Planktothrix agardhii* and *P. rubescens*	–	–	Metcalf and Codd, 2012
Microcystins	*Anabaena, Anabaenopsis, Hapalosiphon, Microcystis, Nostoc, Oscillatoria*, and *Planktothrix*	Cyclic heptapeptide	Inhibition of protein phosphatases (PP1 and PP2A).	Dittmann and Wiegand, 2006
Nodularin	*Nodularia*	Cyclic pentapeptide	Inhibition of protein phosphatases (PP1 and PP2A).	Bagu et al., 1997
Neurotoxins Anatoxin-a	*Anabaena, Aphanizomenon, Cylindrospermum, Oscillatoria, Phormidium*, and *Rhaphidiopsis*	Alkaloid	Binds irreversibly to the nicotinic acetylcholine receptors.	Namikoshi et al., 2003
Anatoxin-a (s)	*Anabaena, Microcystis, Nostoc*, and *Planktothrix*	Guanidine methyl	Inhibits acetylcholinesterase activity.	Matsunaga et al., 1989
β-*N*-methylamino-L-alanine (BMAA)	*Microcystis* and *Planktothrix*	phosphate ester	Causes disorder of Motor system, glutamate agonist, increasing the intracellular concentration of calcium in neurons and inducing neuronal activity by hyperexcitation.	Lobner et al., 2007
Cyanopeptolin	*Anabaena flos-aquae*, and *Anabaena lemmermannii*	–	Transcriptional alterations of genes belonging to DNA damage and repair.	Faltermann et al., 2014
Saxitoxins	*Anabaena, Aphanizomenon, Cylindrospermopsis raciborskii, Lyngbya*, and *Planktothrix*	Carbamate alkaloid	Binds and blocks the sodium channels in neural cells.	Strichartz et al., 1986
Other toxins Debromoaplysiat-oxin	*Lyngbya majuscula*	Polyacetates	Tumor promoters.	Fujiki et al., 1982
Kalkitoxin	*Lyngbya majuscula*	Lipopeptide	Block the sodium channels of nerve cell.	Edwards et al., 2004

Organisms that are eukaryotic algae are designated with “^*^” mark.

These toxins are secreted by cyanobacteria and algae that exert negative impacts on herbivorous zooplanktons (Hansson et al., 2007) and causes serious health hazard by making the water unfit for drinking (Stewart et al., 2006). Among five groups of toxins: hepatotoxin and neurotoxin are the most dangerous to humans as well as animals due to their accumulation in liver and kidney (Wiegand and Pflugmacher, 2005).

## A growing worldwide market for cyanobacterial and algal metabolites

### In cosmetics and other uses

In view of the wide application of algal and cyanobacterial secondary metabolites, photoprotective compounds are being used in several skin care products like anti-aging creams, regenerants, anti-irritant, antioxidants, and anti-inflammatory drugs (Shilpa et al., 2010; Rastogi and Incharoensakdi, 2014; Suh et al., 2014). Some aquatic organisms like *Alaria esculenta* (brown algae), *Ascophyllum nodosum* (brown algae), *Chlorella vulgaris* (green algae), *Chondrus crispus* (red algae), *Dunaliella salina* (green algae), *Mastocarpus stellatus* (red algae), *Nannochloropsis oculata* (algae), and *Spirulina platensis* (blue-green algae) have occupied an important position in the skin care market (Stolz and Obermayer, 2005). *Chlorella* extracts have been used commercially in cosmetics, having collagen stimulating property (Kim et al., 2008). Since last two decades, cases of non-melonoma skin cancer (NMSC) have increased (Halpern and Kopp, 2005) and usage of sunscreen is considered beneficial in these cases (Maier and Korting, 2005) by the health care professionals (Halpern and Kopp, 2005; Seite and Fourtanier, 2008; Diffey, 2009). Due to high demands for safe and best sunscreens in cosmetic industries, exploitation of cyanobacteria has become promising, as MAAs and scytonemin can be used as efficient natural UV blockers in these formulations. They not only prevent damage from the UV radiation but also protect the skin effectively from other problems. These MAAs have absorption maxima in UV range therefore, being used at large scale in various industries (Conde et al., 2000; Whitehead and Hedges, 2005). Some derivatives of MAAs such as tetrahydropyridines have been developed and are applied as sunscreen (Dunlap et al., 1998; Bhatia et al., 2011). Besides this, in paints, plastic, and varnishes industries, MAAs have been widely applied for the manufacturing of photostabilizing agents (Bandaranayake, 1998; Bhatia et al., 2011). Moreover, it was demonstrated that the fusion of two MAAs (shinorine+P334), isolated from red alga *Porphyra umbilicalis*, has suppressed efficiently the negative consequences of UV on human skin (Daniel et al., 2004). A study demonstrated a kinase activity in scytonemin (a photoprotective compound), which may be useful in curing the disorders of proliferation and inflammation (Stevenson et al., 2002). Scytonemin (Garcia-Pichel et al., 1992) prevents up to 90% of solar UV radiation from entering the cell. In addition, scytonemin has antioxidant activity as well as functions as a radical scavenger to prevent cellular damage resulting from ROS produced due to UV-radiation exposure (Matsui et al., 2012; Rastogi et al., 2015). The third most important photoprotective compounds are carotenoids, especially β-carotene, which protects skin against UV-induced photooxidation (Aust et al., 2005; Wertz et al., 2005). Moreover, it has been reported that ketocarotenoid-astaxanthin has vital role in preventing pathological damages in human like photooxidation, inflammation in the cell, prostate and mammary carcinogenesis, aging, ulcers due to *Helicobacter pylori* infection, and skin aging problems (Bennedsen et al., 1999; Guerin et al., 2003; Cardozo et al., 2007). It is proven to be an excellent and more powerful antioxidant than that of vitamins C and E or other carotenoids, while preserving the essential lipids and proteins of human lymphocytes due to its superoxide dismutase and catalase enzyme activities (Bolin et al., 2010; Vílchez et al., 2011). In addition to this, polysaccharides like alginate, fucoidan, and laminaran derived from brown algae, such as *Fucus vesiculosus* and *Turbinaria conoides*, have antioxidative properties (Jea et al., 2009) and can be applicable to prevent skin aging and cutaneous disorders.

Skin whitening has become common tradition all over the world, mainly in Asia (Li E. P. H. et al., 2008). This is because white skin has become a parameter of beauty in Asian culture. In this case, the most common approach for skin whitening is the use of tyrosinase inhibitors (Wang et al., 2011) as the enzyme catalyzes the rate-limiting step of pigmentation. Thomas and Kim (2013) have reported that Fucoxanthin isolated from *Laminaria japonica* suppress tyrosinase activity in melanogenesis in UVB-irradiated mice and UVB-irradiated guinea pigs. Further they have reported that, oral treatment with fucoxanthin suppressed skin mRNA expression linked to melanogenesis, thereby suggesting that fucoxanthin have the capability to negatively regulate the melanogenesis at the transcriptional level. Another brown algal secondary metabolite i.e., Phloroglucinol have tyrosinase inhibitory activity due to their ability to chelate copper (Babitha and Kim, 2011). At industrial scale, they may be widely used in drugs, food additives, and cosmetics (Jha and Zi-rong, 2004). Overall, photoprotective compounds, which do exhibit biological activities, may be used in further research emphasizing their biotechnological applications in order to improve human health. Another important compound agar obtained from algae, has industrial applications in casting, adhesives, coating, printing, dyeing, and culture media (Cardozo et al., 2007). In addition, a unique compound of monoterpenes group-β-phellandrene, made up of 10-carbon has a great commercial potential including personal care, cleaning products, and pharmaceutics (Bentley et al., 2013).

### In defense

Fluctuation in environmental conditions may cause enhancement in ROS production which may damage cells oxidatively. Simultaneously, photosynthetic organisms have developed several strategies to avoid negative consequences of ROS. In this context, PUFAs have been shown to provide protection to the cell against oxidative damage (Kumar et al., 2012). Kumar et al. (2012) have also demonstrated that decline in PUFAs and an enhancement in the activities of antioxidants (i.e., catalase and superoxide dismutase) were sufficient to manage oxidative stress under metal stress. Phycocyanobilins, structurally very close to bilirubin, are regarded as efficient quenchers of different oxygen derivatives (Wagner et al., 1993; Kumar et al., 2016). Therefore, it is thought that phycocyanobilins would have great antioxidant potential since they could protect the living cell against severe oxidative stress (Hirata et al., 2000). Similarly, MAAs may provide protection to the cell by improving the antioxidant status and quenching the superoxide anions and other oxygen derivatives (Suh et al., 2003; De la Coba et al., 2007). From nutrition point of view, a cyanobacterium *Spirulina* can be consumed orally i.e., directly without any processing and is very beneficial to human health including augmentation of the immune system, antioxidant activity, anticancer, and antiviral effects, thereby regulating the hyperlipidemia and cholesterol level, which consequently provide protection to the cell against various disorders such as allergies, obesity, immunomodulation, hepatotoxicity, inflammation, arthritis, and diabetes (Deo et al., 2014; Mishra et al., 2014).

### In biofuels

In the present scenario, energy crisis and global warming have become two burning problems for the human beings. They have occurred due to the disturbance in equilibrium between industrialization, availability of fossil fuel, and population growth. Hence, the identification of alternative and environment friendly renewable energy sources has gained momentum. In this race, presently the algal biofuel has been recognized as a feasible alternative of renewable energy source for sustainable energy production, which has the potential to replace the fossil-based fuels. Cyanobacteria are capable of converting nearly 10% of the solar energy into biomass, while the other algae and energy crops such as sugarcane and corn have the ability to convert only 5 and 1% of solar energy into biomass, respectively. In this way, the photosynthetic prokaryotes like cyanobacteria and microalgae have emerged as useful tools for producing biodiesel, which is cost-effective and eco-friendly to a large extent (Li Q. et al., 2008). The oil obtained from microalgae constitutes 16–68% of dry weight and the yield of oil is recorded up to 136,900 L/ha as compared to the other plant crops, which ranges from 172 to 5950 L/ha (Chu, 2012). Several metabolites like carbohydrates, lipids and fatty acids, important constituents of biofuels, are produced during the Calvin cycle in cyanobacteria and algae. Another process is fermentation, in which a huge amount of carbohydrates can be transformed into bioethanol, fatty acids into acetate, and butyrate into propionate at commercial level. In addition, lipids can also be converted into biodiesel (Parmar et al., 2011; Table 1). Moreover, some green algae like, *Botryococcus* (Rao et al., 2012), *Chlorella* (Münkel et al., 2013), *Scenedesmus* (Xia et al., 2013), *Chlamydomonas* (Nakanishi et al., 2014), *Dunaliella* (Moheimani, 2013), and *Nannochloropsis* (Bartley et al., 2013) may provide raw materials for production of biodiesel. High growth rate, high lipid content, resistant nature under different environmental stimuli and no seasonal limitations on culturing of cyanobacteria and algae make them promising tool for the production of biodiesel at low cost (Chisti, 2007; Ho et al., 2010, 2014). It is necessary to check lipid composition of algae because the content of lipid provides excellency of biodiesel, which can be used for efficient combustion process and also in heating power of engines (Talebi et al., 2013; Wang et al., 2014).

#### Biofuel production

**Figure 8:**
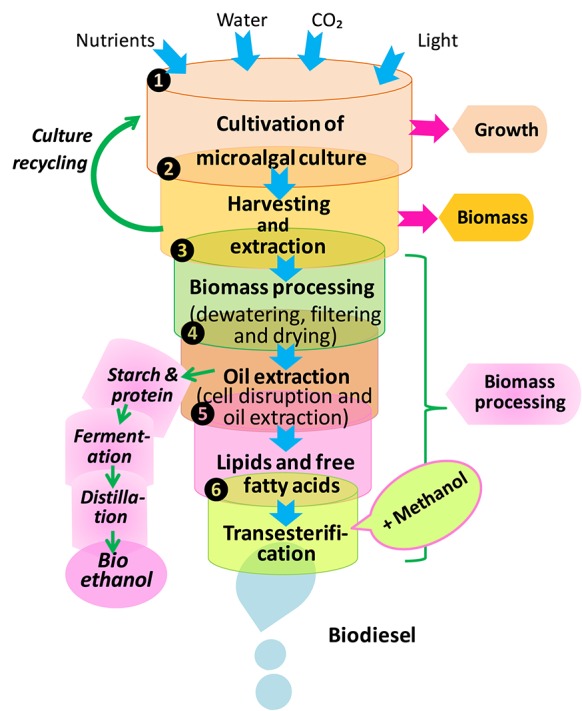
**Processes of biodiesel and bioethanol production from microalgae (modified from Dragone et al., 2010)**.

Biofuel production is a complex process that consists of following stages: (1) microalgae cultivation, (2) harvesting, drying, and cell disruption (cells separation from the growth medium), (3) lipid extraction for biodiesel production through transesterification, and (4) starch hydrolysis, fermentation, and distillation for bioethanol production (Figure 8). There are two major phases for biofuel production i.e., upstream and downstream processes. The upstream stage mainly puts emphasis on different cultivation technologies to maximize biomass quality and quantity, whereas the downstream phase is engaged in harvesting technologies and sustainable biofuel production. So far, various potential sources of biofuel such as biomethane, biohydrogen and bioethanol have been identified for the production of biodiesel. In this way, fatty acids, which have high energy carbon-hydrogen and carbon-carbon bonds, could have a great potential for their application in renewable energy regime either as an additive or major constituent of petroleum (Rupilius and Ahmad, 2006). Several genetically modified cyanobacterial strains e.g., *Anabaena* sp. PCC7120, *Synechococcus elongatus* PCC7942, and *Synechocystis* sp. PCC6803 have been recognized as native producers of hydrocarbons. Furthermore, Tan et al. (2011) have shown that genetically engineered cyanobacterial system may produce various components of biofuels i.e., hydrocarbons and fatty alcohols through photosynthesis. In a study, Liu and Curtiss (2012) have developed a genetic approach termed as “thermorecovery” which helps in liberating free fatty acids that are precursors for biofuel production at commercial level by lysing cultures of cyanobacteria and hydrolysis of membrane lipids.

The major limitations for biofuel production from cyanobacteria and algae are low concentration of biomass and low oil content in the culture. Furthermore, smaller size of microalgae makes them quite costly for harvesting. Moreover, harvesting and drying of algal biomass from high quantity/volume of water are an energy consuming process. The infrastructure and resources needed in the production of large-scale microalgal biomass are costly. The costs of exhaustive management and the equipment for photobioreactor systems are prohibitive, particularly for thousands of hectares of clear containment vessels with accompanying pumps and plumbing needed. In case of large pond, the production appears to be more expensive. Overall, microalgal farming is much more costly and complicated in comparison to the conventional agriculture practices. These difficulties can be overcomed by upgrading the harvesting technologies. Recently, some cost effective technologies have been suggested for microalgal biofuel production:

1. by developing biorefinery or coproduct strategies,
2. by designing high photosynthetic efficient photobioreactors,
3. by developing cost-effective technologies for biomass harvesting and drying,
4. by developing genetic engineering technology to modify the metabolic pathways for microalgal biomass and lipid production and,
5. to understand the symbiotic interactions between microalgae (cyanobacteria and algae) and bacteria, which also affects the biomass as well as lipid production in microalgae.

### Bioethanol

Bioethanol production can be carried out using first, second as well as third generation feedstock. The first generation feedstock includes cereals and legumes like sugar beet, corn, wheat etc. while, the second generation feedstock includes materials rich in lignocellulose like waste or forest residue and the third generation feedstock includes algae. Sugar obtained from molasses, sugarcane, sugar beet is directly fermented by yeast for ethanol production, but they have low conversion costs. It has been suggested that ethanol production from second generation feedstock is more suitable due to less energy requirement and chemical inputs like from 1 ton of sugar beet only 25 gallons (gal) of ethanol is produced, similarly, from 1 ton of sweet sorghum only 20 gallons of ethanol is produced yearly (Sarkar et al., 2012). However, due to their harvesting, purification and treatments needs, their production becomes challenging and less economical, which has shifted the focus toward the third generation feedstock that are easy in cultivation along with high cultivation and less harvesting time. Apart from the convenience in biomass cultivation, the ethanol yield from algal system is very high, which is 5,000–15,000 gal/acre or 46,760–140,290 L/ha, while from sugar beet it is 536–714 gal/acre or 5,010–6680 L/ha, from corn 370–430 gal/acre or 3,460–4,020 L/ha and that from sweet sorghum it is 326–435 gal/acre or 3,050–4,070 L/ha (Chaudhary et al., 2014). The wide variety of algal species such as *Scenedesmus, Chlorella* (Ho et al., 2013), and *Chlamydomonas* (Kim et al., 2006) can accumulate a substantial quantity of carbohydrates in their biomass. Due to the high starch content (ca. 37% dry weight), *Chlorella vulgaris* is a good source of ethanol with 65% conversion efficiency (Hirano et al., 1997). The process for ethanol production involves biomass grounding and then the starch/carbohydrate is converted into sugars, mixed up with yeast and water and kept in large warm tanks called fermenters (Demirbas, 2001). The breakdown of sugar as well as its conversion into ethanol is carried out by yeast (McKendry, 2002). After this, the product undergoes distillation process to remove impurities like water that will be present in the diluted alcohol products (10–15% ethanol). The concentrated ethanol obtained after the process is separated and condensed in liquid form that can be used as petrol substitute (Demirbas, 2001; Machado and Atsumi, 2012). On the other hand, ethanol can also be produced *via* dark fermentation process from microalgae with the maximum productivity of 450 mmol g^−1^ dry weight (Ueno et al., 1998). Moreover, carbohydrates found in cyanobacteria and algae are primarily made up of cellulose (lacks lignin) and starch, which are easily converted to simple sugars for fermentation as compared to the lignocellulosic biomass (John et al., 2011; Ho et al., 2013). Therefore, numerous studies have suggested use of microalgae for biodiesel production that is quite high in comparison to bioethanol production. It seems that in future, production of bioethanol using microalgae will offer a reasonable alternative source (John et al., 2011; Ho et al., 2013).

### In agriculture as biocides

Cyanobacteria and algae are also useful in agriculture sector. Several cyanotoxins, which are derived from cyanobacteria, exhibit various bioactivities and may serve as biocides. These biocides show growth inhibitory response on microorganisms including bacteria, viruses, fungi, and some invertebrates such as crustaceans, bivalves, and also some vertebrates like fish, birds, and mammals (Misra and Kaushik, 1989; Schwartz et al., 1990; Burja et al., 2001). These cyanotoxins have a great potential for developing an active biological compound that could be applied in crop fields as insecticides, herbicides, algicides, and fungicides due to their allelopathic effects (Biondi et al., 2004; Ishibashi et al., 2005; Berry et al., 2008; Rastogi and Sinha, 2009; Table 1). Numerous problems have been reported to arise from application of synthetic pesticides and thus production of biocides with low environmental risk is needed (Isman, 2006). Comparing the ecological impact of synthetic pesticides and biocides, it could be concluded that they have low negative ecological impact and simultaneously maintain growth of producers. Study has demonstrated that cyanotoxins such as microcystins, anatoxin-a and cylindrospermopsin, which are obtained from cyanobacterial strains of *Microcystis, Anabaena*, and *Cylindrospermopsis*, respectively showed greater mortality rate and larvicidal activity (Berry et al., 2008). Thus, application of these cyanotoxins could help in restoring the ecological sustainability (Rastogi and Sinha, 2009).

### In medicine

During the last decades, several bioactive compounds having anti-inflammatory and anticancer property, enzymes and antibiotics have been isolated from cyanobacteria and algae (Burja et al., 2001; Gunasekera et al., 2008; Kwan et al., 2008; Rastogi and Sinha, 2009), which suggest that these organisms may be a great market in developing the important and biotechnologically applicable compounds. Recently, two bioactive compounds viz., dragonamide C and dragonamide D have been isolated from a cyanobacterium *Lyngbya* sp. (Gunasekera et al., 2008) showing anticancerous activity similar to that of recorded with dragonamides, while dragonamide A, B, and E showed *in vitro* activity against leishmaniasis (Jiménez and Scheuer, 2001; McPhail et al., 2007; Balunas et al., 2010). Along with dragonamides, dragomabin was isolated from *Lyngbya* sp. which possesses the best differential toxicity between mammalian cells and parasite. In 2010, Sanchez et al. isolated and identified a series of almiramides A–C from *Lyngbya majuscule which* showed a strong *in vitro* antiparasitic activity against leishmania. Similarly, cryptophycins isolated from *Nostoc* sp. exhibited cytotoxic properties, which provide good opportunities in manufacturing anticancerous drugs (Moore et al., 1996). About twenty-six cryptophycin forms were isolated by Moore group from *Nostoc* sp. GSV 224 (Chaganty et al., 2004). Of the various forms, cryptophycin 52 form was reported to be the most successful and evaluated in phase II clinical trials for curing the platinum resistant ovarian cancer and advanced lung cancer (Edelman et al., 2003; D'Agostino et al., 2006). In other findings, borophycin (polyketide) obtained from cyanobacteria, *Nostoc linckia* and *Nostoc spongiaeforme* exhibited antitumor activity against cancer (Hemscheidt et al., 1994; Torres et al., 2014). Recently, National Cancer Institute (NCR) has announced that a fat soluble photosynthetic pigment, β-carotene is anticarcinogenic in nature. Besides this, it is also effective in reducing the risk of heart diseases by controlling the cholesterol level. Thus, the natural β-carotene can be superior in terms of its anticarcinogen and antiheart disease properties. Due to these desirable medical properties, the demand of natural β-carotene is increasing in the market. Medically *Arthrospira* sp. is very important as it is a rich source of γ-linolenic acid (GLA), which plays vital role in lowering blood pressure by regulating the lipid metabolism.

Omega-3 (ω-3) fatty acids are PUFAs and essential components for the growth of higher eukaryotes (Ward and Singh, 2005). Omega-3 fatty acids are an important structural component of human cell membranes, principally neuronal cells (Brunner, 2006). The use of EPA (eicosapentaenoic acid) and DHA (docosahexaenoic acid) supplements are known to prevent cardiovascular inflammatory conditions (Sijtsma and Swaaf, 2004). In case of cardiovascular health, it is believed that regular consumption of ω-3 fatty acids reduces the risk of cardiac arrhythmia, hypertension, myocardial infarction, and thrombosis because ω-3 fatty acids increase the high-density lipoprotein/low-density lipoprotein (HDL/LDL) ratio thereby decreasing the total cholesterol/HDL ratio (Horrocks and Yeo, 1999). Additionally, omega-3 fatty acids also have positive effect on brain functioning of human beings along with the nervous system (Simopoulos et al., 2009). For the healthy development of fetal brain, the sufficient intake of EPA and DHA is essential in pregnant women (Damude and Kinney, 2008). In infants, for normal growth and functional development, arachidonic acid (ARA), a type of omega-6 fatty acid and DHA are necessary (Dyerberg et al., 1995). Interestingly, an increased DHA consumption may also reduce the severity of depression (Hibbeln and Salem, 1995). The immuno-modulatory effects have been observed (Simopoulos, 1991; Calder, 1996), when they used ω-3 fatty acids in the treatment of inflammatory conditions such as asthma, Crohn's disease, cystic fibrosis, lupus, psoriasis, rheumatoid arthritis, and ulcerative colitis (Simopoulos, 1991; Calder, 1996). According to Hodge et al. (1996) the ingestion of fish oil for more than once a week in children, had a lower probability of suffering from asthma.

Carotenoids are essential for healthy eyes. The two major carotenoids, i.e., lutein and zeaxanthin inhibit photooxidative damage to human retina by accumulating in the macula of retina (Neelam et al., 2005). Astley et al. (2004) showed that occurrence of light mediated diseases can be reduced by antioxidant activity of carotenoids. In addition, several epidemiological evidences show that high dietary intake of carotenoids decrease the risk of cancer, as lycopene has been suggested to be effective against prostate cancer (Ben-Dor et al., 2005).

The highly purified agar (also called agarose in neutral fractions) is used as anticoagulants, bulking agents, capsules, laxatives, suppositories, and tablets, which are useful from pharmaceutical point of view (Cardozo et al., 2007). Moreover, agar is employed for cancer cell therapy because it can persuade the apoptosis of these cells *in vitro* (Chen et al., 2004).

Algae are good sources of lectins that have specific role in advanced medical sciences. Some examples are blood group typing and definition of secretor status, detection of disease-related alterations of glycan synthesis, quantification of aberrations of cell surface glycan presentation and malignancy (Rudiger and Gabius, 2001). Besides this, they deliver vaccines across the mucosal surfaces and binds there due to their bioadhesive property (Jepson et al., 2004). A detail outline for use of cyanobacteria and algae as medicine is given in Table 4.

**Table 4:** **Various therapeutic agents produced by cyanobacteria and algae and their potential uses**.

**Compound(s)**	**Organism**	**Activity**	**References**
Acetylated sulfoglyco-lipids	*Oscillatoria raoi*	Antiviral	Reshef et al., 1997
Acutiphycin	*Oscillatoria acutissima*	Anticancer	Barchi et al., 1984
Aeruginosins	*Microcystis, Nodularia*, and *Oscillatoria*	Serine proteases inhibitor	Shin et al., 1997
Agardhipeptin	*Oscillatoria agardhii*	Enzyme inhibitor	Luukkainen et al., 1993
Alkaloids (ambiguine H & I isonitriles)	*Fischerella* sp.	Antibacterial	Raveh and Carmeli, 2007
Allophycocyanin	Cryptomonads^*^	Enterovirus 71	Shih et al., 2000
Ambigol A, B	*Fischerella ambigua*	antifungal, antibacterial	Falch et al., 1993
Ambiguine I isonitrile	*Fischerella* sp. (*Fischerella ambigua*)	Antibacterial	Raveh and Carmeli, 2007
Anatoxin-a	*Anabaena* sp.	Larvicide	Berry et al., 2008
Anatoxin-a	*Anabaena circinalis*	Inflammatory	Rajeev and Xu, 2004
Anhydrohapaloxindole	*Hapalosiphon fontinalis*	Antifungal	Moore et al., 1987
Ankaraholide A	*Lyngbya majuscula*	Anticancer	Mynderse et al., 1977
Aplysiatoxin	*Geitlerinema*	Anticancer	Andrianasolo et al., 2005
Aponin	*Gomphosphaeria aponina*	Antialgal	Bhadury and Wright, 2004
Apratoxin A	*Lyngbya majuscula*	Anticancer	Andrianasolo et al., 2005
Apratoxins B–C	*Lyngbya* sp.	Anticancer	Luesch et al., 2002
Apratoxin D	*Lyngbya majuscula* and *Lyngbya sordida*	Anticancer	Gutierrez et al., 2008
Apratoxin E	*Lyngbya bouilloni*	Anticancer	Matthew et al., 2008
Apratoxins F and G	*Lyngbya bouilloni*	Anticancer	Tidgewell et al., 2010
Aurilide B	*Lyngbya majuscula*	Anticancer	Han et al., 2006
Aurilide C	*Lyngbya majuscula*	Anticancer	Han et al., 2006
Bastadin	*Anabaena basta*	Antibiotic	Miao et al., 1990
Bauerines A–C	*Dichotrix baueriana*	Anti-Herpes simplex virus type 2 (HIV-2)	Larsen et al., 1994
Belamide A	*Anabena variabilis*	Antibiotic	Ma and Led, 2000
Bis-(χ-butyrolactones)	*Symploca* sp.	Anticancer	Simmons et al., 2006
Bisebromoamide	*Lyngbya* sp.	Anticancer	Teruya et al., 2009
Biselyngbyaside	*Lyngbya* sp.	Anticancer	Teruya et al., 2009
Borophycin	*Nostoc linckia* and *Nostoc spongiaeforme*	Anticancer	Hemscheidt et al., 1994
BP-1 Thermostable polyphosphate kinase	*Thermosynechococcus elongatus*	Production of dipeptides	Sato et al., 2007
Butanoic acid and Methyl lactate	*Haematococcus pluvialis*	Antibacterial	Santoyo et al., 2009
Calcium spirulan	*Spirulina platensis*	Antiviral (Anti-(HIV) Human Immunodeficiency Virus)	Hayashi et al., 1996
Calophycin	*Calothrix fusca*	Fungicide	Moon et al., 1992
Calothrixins A,B	*Calothrix* sp.	Antimalarial, anticancer	Bernardo et al., 2007; Khan et al., 2009
Carazostatin	*Hyella caespitose*	Antifungal	Burja et al., 2001
Carbamidocyclophanes A-E	*Nostoc* sp.	Antibiotic and cytotoxic	Bui et al., 2007
Carmabin A,B	*Lyngbya majuscula*	Antimalarial, anticancer, antiproliferative	McPhail et al., 2007
Caylobolide A	*Lyngbya majuscula*	Anticancer	MacMillan and Molinski, 2002
Caylobolide B	*Phormidium* sp.	Anticancer	Salvador et al., 2010
Circinamide	*Anabaena circinalis*	Enzyme inhibitor	Negri and Jones, 1995
Coibamide A	*Leptolyngbya* sp.	Anticancer	Medina et al., 2008
Cryptophycins	*Nostoc* sp.	Anticancer	Moore et al., 1996
Curacin A	*Lyngbya* sp.	Anticancer	Simmons et al., 2005
Curacin A	*Lyngbya majuscula*	Microtubulin assembly inhibitors	Shimizu, 2003
Cyanobactericin	*Scytonema hofmanni*	Antialgal	Abarzua et al., 1999
Cyanobacterin LU-1	*Nostoc linckia*	Antialgal	Gromov et al., 1991
Cyanovirin -N	*Nostoc ellipsosporum*	Anti-HIV, antiviral	Dey et al., 2000
Cyclic polypeptide	*Lyngbya majuscula*	Anti-HIV activity	Rajeev and Xu, 2004
Cylindrospermopsin	*Cylindrospermopsis* sp.	Larvicide	Berry et al., 2008
Debromoaplysiatoxin	*Lyngbya majuscula*	Inflammatory	Shimizu, 2003
Didehydromirabazole	*Scytonema mirabile*	Antibiotic	Stewart et al., 1988
Diterpenoid	*Nostoc commune*	Antibacterial	Asthana et al., 2009
Dolastatins	*Lyngbya* sp. and *Symploca* sp.	Antimalarials, anticancer	Fennell et al., 2003; Catassi et al., 2006
Dragonamide A, B	*Lyngbya majuscula*	Antimalarial	McPhail et al., 2007
Dragonamide C, D	*Lyngbya polychroa*	Anticancer	Gunasekera et al., 2008
Eicosapentaenoic acid	Phaeodactylum tricornutum^*^	Antibacterial	Smith et al., 2010
Ester	*Navicula delognei*	Antibacterial	Findlay and Patil, 1984
Ethyl Tumonoate A	*Oscillatoria margaritifera*	Anticancer	Engene et al., 2011
Extracellular sulfated polysaccharides	Cochlodinium polykrikoide^*^	Influenza virus A and B, RSV A and B, and HSV-1	Hasui et al., 1995
Fatty acids (coriolic acid and α-dimorphecolic acid	*Oscillatoria redekei*	Antibacterial	Mundt et al., 2003
Fischambiguine B	*Fischerella ambigua*	Antibacterial	Mo et al., 2010
Fischerindole L	*Fischerella muscicola*	Antifungal	Park et al., 1992
Fisherellin	*Fischerella muscicola*	Antialgal, antifungal	Dahms et al., 2006
Galactosyldiacylglycerols	*Phormidium tenue*	Antialgal, anti-HIV	Rajeev and Xu, 2004
Gambieric acids A and B	Gambierdiscus toxicus^*^	Antifungal	Bhadury and Wright, 2004
γ- linolenic acid	*Spirulina platensis*	Predecessor of arachidonic acid	Cohen, 1999
Grassypeptolide	*Lyngbya confervoides*	Antiproliferative	Kwan et al., 2008
γ-linolenic acid	*Fischerella* sp.	Antibacterial	Asthana et al., 2006
Goniodomin A	Goniodoma pseudogoniaulax^*^	Antifungal	Bhadury and Wright, 2004
Hapalindole	*Hapalosiphon fontinalis*	Antifungal	Burja et al., 2001
Hierridin B	*Cyanobium* sp.	Antitumor	Leão et al., 2013
Hoiamide A	Assemblage of *Lyngbya majuscule* and *Phormidium* gracile	Anticancer	Choi et al., 2010
Hoiamide B	Cyanobacterial sample	Anticancer	Choi et al., 2010
Homodolastatin 16	*Lyngbya majuscula*	Anticancer	Davies-Coleman et al., 2003
Hormothamnins	*Hormothamnion enteromorphoides*	Antibacterial, antifungal	Gerwick et al., 1989
Ichthyopeptins A and B	*Microcystis ichthyoblabe*	Antiviral	Zainuddin et al., 2007
Indolocarbazoles	*Nostoc sphaericum*	Antiviral	Cohen, 1999
Isomalyngamide A and A-1	*Lyngbya majuscula*	Anticancer	Chang et al., 2011
Kaempherol	*Gracilaria dendroides*	Anticancer	Al-Saif et al., 2014
Kalkitoxin	*Lyngbya majuscula*	Sodium channel blocker	Shimizu, 2003
Karatungiols	Amphidinium^*^ sp.	Antifungal	Washida et al., 2006
δ-lactone malyngolide	*Lyngbya majuscula*	Antibacterial	Cardllina et al., 1979
Kawaguchipeptin B	*Microcystis aeruginosa*	Antibacterial	Dahms et al., 2006
Lagunamide C	*Lyngbya majuscula*	Anticancer	Tripathi et al., 2011
Largazole	*Symploca* sp.	Anticancer	Zeng et al., 2010
Laxaphycins	*Anabaena laxa*	Antifungal	Frankmölle et al., 1992
Lyngbyastatin	*Lyngbya confervoides*	Serine protease inhibitor	Matthew et al., 2007
Lyngbyatoxins	*Lyngbya majuscula*	PKC activator	Shimizu, 2003
Majusculamide C	*Lyngbya majuscula*	Anticancer	Pettit et al., 2008
Malevamide D	*Symploca hydnoides*	Anticancer	Horgen et al., 2002
Malyngamide 2	*Lyngbya sordida*	Anticancer	Malloy et al., 2011
Methanolic and hexanolic extracts	Chlamydomonas reinhardtii^*^	Antibacterial	Ghasemi et al., 2007
Microcystin	*Microcystis aeruginosa*	Algicide/larvicide/herbicide	Berry et al., 2008
Muscoride	*Nostoc muscorum*	Antibiotic	Nagatsu et al., 1995
Muscoride A	*Nostoc muscorum*	Antibacterial	Nagatsu et al., 1995
Naienones A-C	*Synechocystis* sp.	Antitumoural	Nagle and Gerwick, 1995
Norharmane	*Nostoc insulare*	Antibacterial	Volk and Furkert, 2006
Noscomin	*Nostoc commune*	Antibacterial	Jaki et al., 1999
Nostocarboline	*Nostoc* sp.	Antimalarial, antileishmaniasis, cholinesterase inhibitor	Barbaras et al., 2008
Nostocine A	*Nostoc spongiaeforme*	Antibiotic	Hirata et al., 2003
Nostocyclamide	*Nostoc* sp.	Antifungal	Moore et al., 1988
Nostocycline A	*Nostoc* sp.	Antibacterial	Ploutno and Carmeli, 2000
Nostodione	*Nostoc commune*	Antifungal	Bhadury and Wright, 2004
Nostoflan	*Nostoc flagelliforme*	Antiviral	Hayashi et al., 2008
p-KG03exopolysaccharide	Gyrodinium impudicum^*^	Encephalomyocarditis virus	Yim et al., 2004
Pahayokolides	*Lyngbya* sp.	Antialgal/larvicidal	Gantar et al., 2008
Parsiguine	*Fischerella ambigua*	Antibacterial	Ghasemi et al., 2004
Peptide	Stichochrysis imobilis^*^	Antibacterial	Berland et al., 1972
*Phaeocystis* sp. acrylic acid	Phaeocystis^*^ sp.	Antibacterial	Sieburth, 1960
Pheophorbide α-,β-like compounds	Dunaliella primolecta^*^	HSV-1	Ohta et al., 1998
Palmyramide A	*Lyngbya majuscula*	Anticancer	Taniguchi et al., 2010
Phytoalexin	*Scytonema ocellatum*	Antifungal	Patterson and Bolis, 1997
Pitipeptolides C	*Lyngbya majuscula*	Anticancer	Montaser et al., 2011a
Pitiprolamide	*Lyngbya majuscula*	Anticancer	Montaser et al., 2011b
Polysaccharide	Navicula directa^*^	HSV-1 and -2, Influenza A virus	Lee et al., 2006
Polyether compounds	Prorocentrum lima^*^ and Dinophysis fortii^*^	Antifungal	Bhadury and Wright, 2004
Quercetin	*Gracilaria dendroides*	Antifungal	Al-Saif et al., 2014
Radiosumin	*Plectonema radiosum*	Enzyme inhibitor	Mooberry et al., 1995
Rutin	*Gracilaria dendroides*	Enzyme inhibitor	Al-Saif et al., 2014
Schizotrin A	*Schizothrix* sp.	Antifungal, antibacterial	Pergament and Carmeli, 1994
Scytophycins	*Scytonema pseudohofmanni*	Antifungal	Burja et al., 2001
Scytophycins	*Scytonema* sp., *Tolypothrix* sp.	Antifungal	Ishibashi et al., 1986; Carmeli et al., 1990
Scytoscalarol	*Scytonema* sp.	Antibacterial	Mo et al., 2009
Scytovirin	*Scytonema varium*	Anti-HIV activity	Bokesch et al., 2003
Somocystinamide A	*Lyngbya majuscula*	Anticancer	Wrasidlo et al., 2008
Spirulan	*Spirulina platensis*	Antiviral	Hayashi et al., 1996
Sulfolipids	*Lyngbya lagerhimii* and *Phormidium tenue*	Anti-HIV activity	Rajeev and Xu, 2004
Sulfated polysaccharides	Chlorella autotrophica^*^ and Ellipsoidon^*^ sp.	Viral hemorrhagic septicemia virus, African swine fever virus	Fábregas et al., 1999
Symplocamide A	*Symploca* sp.	Antimalarial, antileishmaniasis, anticancer	Linington et al., 2008
Symplostatin 3	*Symploca* sp.	Anticancer	Luesch et al., 2002
Tanikolide	*Lyngbya majuscula*	Antifungal	Singh et al., 1999
Tenuecyclamides	*Nostoc spongiaeforme*	Antibacterial and cytotoxic	Banker and Carmeli, 1998
Thermostable enzymes	*Phormidium* sp.	Catalysis of reactions	Piechula et al., 2001
Tjipanazoles	*Tolypothrix tjipanasensis*	Anticancer	Bonjouklian et al., 1991
Tolybyssidins	*Tolypothrix byssoidea*	Antifungal	Jaki et al., 2001
Tolyporphin	*Tolypothrix nodosa*	Antibiotic	Prinsep et al., 1992
Tolytoxin	*Scytonema ocellatum*	Antifungal	Patterson and Carmeli, 1992
Toyocamycin	*Tolypothrix tenuis*	Antifungal	Banker and Carmeli, 1998
Tubercidin toyocamycin	*Plectonema radiosum* and *Tolypothrix tenuis*	Fungicidal, cytotoxic	Stewart et al., 1988
Venturamide A,B	*Oscillatoria* sp.	Antimalarial	Linington et al., 2007
Veraguamides A-G	*Symploca cf. hydnoides*	Anticancer	Mevers et al., 2011
Wewakazole	*Lyngbya sordida*	Anticancer	Malloy et al., 2011
Wewakpeptins	*Lyngbya semiplena*	Anticancer	Han et al., 2005

Organisms that are eukaryotic algae are designated with “^*^” mark.

### In food and food colorant

Among diverse metabolites procured from cyanobacteria and algae, fatty acids particularly PUFAs have gained much consideration due to their nutritional importance. Cyanobacteria and algae produce an enormous amount of PUFAs and thus, are contributing in manufacturing of fats and oils at commercial level as alternative sources of animal and plants' oil. Apart from this, PUFAs especially EPA and DHA, are being implicated in the prevention of cardiovascular disease. The oil extracted from *Crypthecodinium cohnii* contains 40–50% DHA but no EPA or other long chain poly unsaturated fatty acids (LC-PUFA) and DHA is very important for brain and eye development in infants (Kroes et al., 2003; Ward and Singh, 2005). Since, in an aquatic ecosystem fishes and other herbivores have lesser capability of producing PUFAs, they obtain them from cyanobacteria and microalgae, which are rich sources of different kinds of fatty acids. Property of excess fatty acid production by cyanobacteria and microalgae makes them suitable candidates for aquaculture (Tonon et al., 2002; Guedes et al., 2011). The microalgal fatty acids consisted of triacylglycerides (TAG), a class of lipid mainly used by oleaginous eukaryotic micro-organisms for storage of their fatty acids under stress conditions (Ratledge, 2004). TAG offers the option to partially replace the functions of currently used vegetable oils. For example, the presence of alpha-linolenic and linoleic acid may partially replace the essential fatty acid contribution from rape seed and sunflower oils. The presence of LC-PUFA such as arachidonic acid, EPA, DHA, and stearidonic acid are of great interest in the evaluation of nutritional composition of an algal species to be used as food for marine organisms (Mozaffarian and Rimm, 2006; Harris et al., 2008). An alga, *Gracilaria verrucosa* belonging to the family rhodophyceae is one of the most exploited red seaweeds of Chilika, India and is usually known for its utilization in food industries (Gouda et al., 2013). Riahi et al. (2011) have reported that covering of mushroom growing fields with cyanobacterial cultures enhances yield, dry weight, and protein content of mushroom due to the secretion of plant growth regulators like auxins, sugars, and vitamins. Moreover, the cyanobacterium *Arthrospira platensis* has been reported as a rich source of protein [therefore regarded as single cell protein (SCP)], fatty acids, and feed supplements (Mishra et al., 2014). It is thought that the frequent use of *Spirulina* in diet may encourage the health of patients suffering from malnutrition, immune-suppression, hepatic, and neural compromise but further exploration on the antiviral impacts of this alga and its pharmaceutical applications are needed (Deo et al., 2014; Mishra et al., 2014). Due to high nutritional supplements for humans as well as animals, green algae i.e., *Chlorella vulgaris, Dunaliella salina, Haematococcus pluvialis*, and cyanobacterium i.e., *Spirulina maxima* are relevant in biotechnological fields. *Spirulina platensis*, due to its enrichment in pigments (Madhyastha and Vatsala, 2007), PUFAs (Sajilata et al., 2008), proteins (Colla et al., 2007; Kumar et al., 2014), vitamins, and phenolics (Ogbonda et al., 2007) had become a trademark of nutritional supplements. *Chlorella* is another example, which is gaining worldwide attention, because of its high demand in health food stores (Hills and Nakamura, 1978).

Besides this, the commercial importance of microalgal pigments cannot be avoided as they have become a necessary part of natural food colorant. β-carotene, which is obtained from some microalgae, is used as a food additive for enhancing: (i) coloration of fish flesh and egg yolk (specifically provides yellow color to margarine) and (ii) fertility and health of grain-fed cattle (Borowitzka, 1988). β-carotene is also used in cosmetics and food products like margarine, cheese, fruit juices, baked goods, dairy products, canned goods, and confectionary (Dufosse et al., 2005). Although, it is non-photostable and color bleach in cooking, still it has vastly gripped a potential market for microalgae-derived food colorant. β-carotene, is naturally obtained from green alga *Dunaliella salina*, which constitutes 14% of its dry weight (Metting, 1996) and the antioxidant activity of β-carotene from *Dunaliella* is much higher than that of synthetic one. Another carotenoid, astaxanthin is produced by the green alga *Haematococcus pluviali*, which reaches up to 4–5% of dry weight and the market of astaxanthin is worth US $200 million with an average price of US $2,500/kg.

Carrageenan is obtained from macroalga, *Kappaphycus alvarezii* and supplied on a large scale in food industry. It is generally used as emulsers/stabilizers, due to their thickening and suspension forming properties in numerous foods, especially milk-based food products like ice cream, chocolate milk, puddings, jellies, evaporated milk, jams, salad dressings, dessert gels, pet foods, and meat products. Apart from their usage in foods, they are also used in medicine for their anticoagulant, antitumor, antiviral, and immunomodulation activities (Schaeffer and Krylov, 2000; Zhou et al., 2005). The agar has also its importance in preparation of gel substrate in biological culture media. Besides, these compounds, some cyanobacteria and alage are also a rich source of amino acids. For instance, *Nostoc flagelliforme* contains 19 amino acids and out of these eight are essential one for human and the production of these essential amino acid is 35.8–38.0% of the total amino acid (Han et al., 2004).

### Polyhydroxyalkanoates (PHAs): a substitute for non-biodegradable plastics

In the past few decades, the enormous uses of non-biodegradable plastics by humans have stressed almost the whole ecosystem especially in developing countries like India. The properties of PHAs are comparable to that of polypropylene (Doi, 1990; Loo and Sudesh, 2007), have attracted the attention of scientists as they are potential substitutes for non-biodegradable petrochemical-based plastics.

Microorganisms usually assimilate and store nutrients when there is high nutrient availability in surroundings. Among these stored nutrients, the lipoidic materials i.e., PHAs are accumulated in excess carbon availability (Anderson and Dawes, 1990; Nikodinovic-Runic et al., 2013). After assimilation of these carbons, they are processed biochemically and converted into monomer units (hydroxyalkanoate) and thereafter are polymerized and stored in the cell cytoplasm in form of water insoluble granules. It has been reported that two cyanobacterial strains *Spirulina platensis* and *Synechocystis* sp. accumulate ~6–7% hydroxyalkanoateon on dry weight basis (Campbell et al., 1982; Sudesh, 2004). Most commonly synthesized PHA by alga is poly 3-hydroxybutyrate (PHB). Since the biosynthesis efficiency for PHB in cyanobacteria is quite low therefore, in order to increase the production, PHB biosynthetic gene is introduced from bacterium *Ralstonia eutropha* into *Synechococcus* 7942 along with nitrogen starvation and acetate supplementation condition and production reached upto 25.6% of the dry cell weight (Takahashi et al., 1998). The metabolite production which is being enhanced by engineering the cyanobacteria has been discussed in the following section.

## Genetically modified organisms (GMO) and metabolite production

The high amount of metabolite production from cyanobacteria has compelled the scientists to engineer these organisms in order to obtain maximum production. Several metabolites like alcohols, fatty metabolites (fatty acid, fatty alcohol, and fatty hydrocarbon), hydrocarbon (ethylene), carbohydrates (mannitol, lactate, and Glucosylglycerol), carboxylic acid, and terpenes obtained from cyanobacteria are applicable at commercial level and therefore to enhance their production, cyanobacteria and algae are being engineered (reviewed by Oliver et al., 2016). Likely, engineered *Synechococcus elongatus* PCC 7942 has 1.8-folds higher production of 2,3-butanediol (23BD) than that of the parent strain (Oliver et al., 2013). The maximum 23BD production was ~22 mg/L/h. Similar to this, Hirokawa et al. (2016) constructed a 1,3-propanediol biosynthetic pathway in *Synechococcus elongatus* PCC 7942 and observed that its average productivity was 0.9 mg/L/h, which constituted 288 mg/L after 14 days. Likewise, ethanol production was enhanced 83% (productivity 11 mg/L/h) after engineering the pyruvate carboxylase enzyme in *Synechocystis* sp. PCC 6803 (Luan et al., 2015). The production of ethylene, an important component of polymers has been enhanced by engineering the *Synechococcus elongatus* PCC 7942 and *Synechocystis* sp. PCC 6803 and the production was improved by 64% (0.9 mg/L/h; Takahama et al., 2003; Ungerer et al., 2012; Lee et al., 2015). As the need of biofuel production is increasing, therefore, to optimize free fatty acids production in *Synechococcus elongatus* PCC 7942 and *Synechocystis* sp. PCC 6803, the alternative carbon sinks were removed as well as flux was increased for fatty acid biosynthesis, which gave the productivity of 0.1 and 0.4 mg/L/h, respectively (Liu et al., 2011; Ruffing and Jones, 2012). The production of fatty alcohol and fatty hydrocarbons has also been improved by employing the same engineering process. Similar to fatty acids, the production of carbohydrates, carboxylic acids, and terpenes obtained from cyanobacteria has been incresaed using the engineering process, which has been discussed in detail in review by Oliver et al. (2016). Regarding the genetic manipulation in the case of algae, some experiments have been performed with *Chlamydomonas reinhardtii* but no successful results have been obtained and this engineering process needs to be rectified in the case of algae.

## Algal bioprocessing and challenges

As cyanobacteria and algae are a renewable source of drop-in fuels, feeds, fertilizers, nutritional oils, and pharmaceuticals. They can also provide waste water treatment and other remediation services and many more new applications are continuously being discovered. All these applications have to be commercialized and algal bioprocessing has been put forward as a flagship technology for driving the products or other valuable chemicals that are obtained from cyanobacteria and algae. The commercialization programme is being performed for harnessing the unequaled potential of algae to provide us with sustainable products, drive economic growth, and reduce greenhouse gas emissions. Several companies like solazyme, algenol, terra biologics etc. are heading toward this agenda but having some key points that have to be addressed by Algae biomass organization (ABO) that include: (i) production of renewable fuel like ethanol, gasoline, diesel, and jet fuel and energy, (ii) production of more protein, feed, and oil, (iii) health and nutrition and (iv) materials and services. Outline of some companies has been addressed in the following paragraphs.

Algenol is a biotechnology company which is involved in commercializing the algae technology that had been patented for production of ethanol, gasoline, jet, and diesel fuel for a targeted cost of $1.30 per gallon using algae, sunlight, carbon dioxide, and salt water. The ethanol obtained from patented strain is 20 times more than that of corn ethanol. The current yield is 8,000 gallons /acre/year. The production is carried out in fully closed and sealed photo bioreactors and the waste algae are converted to diesel, jet fuel, and gasoline using hydrothermal liquefaction.

Global Algae Innovations is another company which uses low cost algae production technologies. The company uses suite of algae grown in open ponds with novel, low-cost production technology in every process step. As a result, economical, sustainable production of protein and biofuel are now within reach. It also leverages the production of other algae markets such as functional foods, nutraceuticals, pigments, and aquaculture. The harvest technology of this company has 100% efficiency.

TerraVia Holdings (formerly Solazyme) is also a Biotechnology company which uses the copyrighted technology of converting low-cost plant-based sugars into high-value oils. This company shifted its focus from bio-fules to sustainable food oils and personal care products in March, 2016. However, commercialization processes have some challenges that have been discussed in detail in review by Griffiths et al. (2012). The challenges to be addressed have been briefly outlined below:

1. Increasing productivity in large-scale outdoor microalgal culture.
2. Minimizing contamination by predators and other algal species.
3. Mitigating temperature changes and water loss due to evaporation.
4. Optimizing supply of light and CO_2._
5. Developing cheap and efficient reactor designs.
6. Developing cost and energy-efficient methods of harvesting dilute suspensions of small microalgal cells.
7. Decreasing the overall energy and cost requirements, particularly for pumping, gas transfer, mixing, harvesting, and dewatering.
8. Improving resource utilization and productivity through a biorefinery approach.
9. Producing valuable co-products.
10. Decreasing environmental footprint through recycling of water, energy, and nutrients.

## Conclusions and future perspective

Since the beginning of the civilization, biologically active compounds, which are obtained from diverse range of algae and cyanobacteria have been widely explored. Cyanobacteria and algae are rich sources of various compounds including pigments, lectins, fibers, halogenated compounds, steroids, antioxidants, vitamins, polyketides, polysaccharides, MAAs, proteins, and essential lipids. Therefore, they are widely used in different countries due to their multifunctional applications in nutraceuticals as well as in pharmaceuticals. Cyanobacterial and algal secondary metabolites possess several pharmaceutical applications such as antiviral, anticancer, and antimicrobial activities. Wide use of biocides has emerged as eco-friendly tactic as they are easily degradable in nature as compared to other synthetic pesticides. Undoubtedly, in the past few decades, our understanding in the field of algal metabolites has significantly improved, but there are still many steps we have to reach. We are entering in the blooming era of cyanobacteria and algae, our stage is set and it is the time, we uncover the enigma of cyanobacterial and algal metabolites. Definitely, by uncovering novel functions of algal secondary metabolites a new scenario will appear with specific reflection to humanity. This review has emphasized that cyanobacteria and algae are promising sources of structurally diverse biologically active compounds such as terpenes, alkaloids, steroids, polysaccharides, lipids, and polyphenolics which have several utilities in various industries. Nevertheless, further investigations are required for compiling secondary metabolites profile of cyanobacteria and algae in order to make them more useful for human welfare. There is a need to find out how we can convert the present days technology into a green technology for exploiting these cyanobacteria and algae. We should also think upon the strategy for disseminating this commercialization at small scale as well as at large scale. There is a need to find out answer to these questions like whether the production rate of metabolites is sufficient to meet out the demands in comparison to plants? Whether these metabolites could have some more beneficiary roles? Whether the changes made by bioenegineering could be employed in plant system to enhance the production from both cyanobacteria/algae and plants? Future work will no doubt reveal novel functions for secondary metabolites and the future research in this area will be very promising.

## Author contributions

RS, PP, SS, JK, and MS prepared draft of this review and wrote it. AB, VS, and SP corrected and finalized review.

### Conflict of interest statement

The authors declare that the research was conducted in the absence of any commercial or financial relationships that could be construed as a potential conflict of interest.
